# Reconsideration of *r/K* Selection Theory Using Stochastic Control Theory and Nonlinear Structured Population Models

**DOI:** 10.1371/journal.pone.0157715

**Published:** 2016-06-23

**Authors:** Ryo Oizumi, Toshikazu Kuniya, Yoichi Enatsu

**Affiliations:** 1 Director General Office for Policy Planning and Evaluation, Ministry of Health, Labour and Welfare Japan, 1-2-2, Kasumigaseki, Chiyoda-ku, Tokyo 100-8916, Japan; 2 Graduate School of System Informatics, Kobe University, 1-1 Rokkodai-cho, Nada-ku, Kobe 657-8501, Japan; 3 Department of Mathematical Information Science, Tokyo University of Science 1-3 Kagurazaka, Shinjuku-ku, Tokyo 162-8601, Japan; Centre for Computational Biology and Evolution, CHINA

## Abstract

Despite the fact that density effects and individual differences in life history are considered to be important for evolution, these factors lead to several difficulties in understanding the evolution of life history, especially when population sizes reach the carrying capacity. *r*/*K* selection theory explains what types of life strategies evolve in the presence of density effects and individual differences. However, the relationship between the life schedules of individuals and population size is still unclear, even if the theory can classify life strategies appropriately. To address this issue, we propose a few equations on adaptive life strategies in *r*/*K* selection where density effects are absent or present. The equations detail not only the adaptive life history but also the population dynamics. Furthermore, the equations can incorporate temporal individual differences, which are referred to as internal stochasticity. Our framework reveals that maximizing density effects is an evolutionarily stable strategy related to the carrying capacity. A significant consequence of our analysis is that adaptive strategies in both selections maximize an identical function, providing both population growth rate and carrying capacity. We apply our method to an optimal foraging problem in a semelparous species model and demonstrate that the adaptive strategy yields a lower intrinsic growth rate as well as a lower basic reproductive number than those obtained with other strategies. This study proposes that the diversity of life strategies arises due to the effects of density and internal stochasticity.

## Introduction

Density effects in life history are considered to be important for evolution. Intraspecific competition has led to the evolution of antlers and peacock feathers in sexual selection, and another type of selection has led to phase variation, depending on the population density, in migratory locusts [1]. Determining what life strategies can evolve in each population size is a difficult question; these questions describe an area of research referred to as *r*/*K*-selection theory. This theory has captured the attention of ecologists for several decades. It was proposed in MacArthur and Wilson’s book [2], which discussed it in the context of the differences between adaptive reproductive strategies at low and high levels of intraspecific competition. At the lower levels, such as an environment affected by disturbance (*r*-selection), life histories evolve to maximize the intrinsic rate of natural increase (IRNI), whereas at the higher levels, such as saturated populations (*K*-selection), life histories evolve to maximize the carrying capacity. These different life histories were classified by Pianka [3]. For instance, species affected by *r*-selection have precocity and prolificacy. Conversely, the other selection involves altricity in addition to producing a smaller number of offspring. According to Reznick *et* *al* (2002), the classifications and theory have been discussed in many papers, with both agreement and disagreement, since the theory was first published [4]. The papers that disagree with the theory state that several species do not seem not to match Pianka’s classification, e.g., trees with both longevity and prolificacy. To clarify the issue, researchers have developed many precursor theoretical models and approaches, such as optimal life schedule problem (OLSP), adaptive dynamics (AD), structured demographic models (SDM), and so on. The paradigm of *r*/*K* selection theory has shifted to a theory of density dependence of mortality in SDMs. Many studies are based on the hypothesis that all intraspecific competition influences fertility and mortality. Furthermore, a recent work extended these models to adapting environmental stochasticity in addition to density effects [5]. Ecologists speculate that adaptive life histories under both selections evolve as the consequence of density and environmental effects.

One reason why *r*/*K* selection theory has attracted the attention of ecologists is that there are several difficulties concerning definitions in *K*-selection; it is not well understood what individuals should maximize (or minimize) in the selection. Because the basic reproductive number $R_{0}$ is always one for individuals in saturated populations, it is difficult to find factors augmenting their carrying capacity on the individual scale. AD postulates that adaptive species have an evolutionarily stable strategy (ESS) that cannot be disrupted by any other mutant strategies. The analysis of AD requires appropriate replicator dynamics providing a fitness function (or invasion fitness). The fitness function does not necessarily clarify the relationship with individual life dynamics such as size growth, reproduction, death, and so on. Age-SDMs can be applied to this problem [6, 7]. The age-SDMs define carrying capacity as the interior equilibrium generated by the density effects on generations in terms of fertility and mortality. This model uses a characteristic function incorporating fertility and mortality functions with respect to age that includes IRNI and $R_{0}$ as the fitness function. Though it is reasonable, the model deals with intraspecific competitions in terms of only fertility and mortality. For instance, competition for food intake may inhibit growth and maturity for each individual. The inhibition will reduce IRNI, even if $R_{0}$ is preserved. To handle the wide range of life history evolutions, age-SDMs are not sufficient; they should take into account other ingredients (e.g., size, spatial position, genetic expressions, and individual differences). Given the components of life history, the theory associated with *r*/*K*-selection should provide an appropriate definition of adaptive strategy in the context of carrying capacity. Therefore, a theoretical framework is required that describes the population dynamics and life schedule of individuals simultaneously.

These problems mainly lack a theory that connects individuals and the population scale. Recent works have addressed this issue. Oizumi and Takada (2013) derived a characteristic function that incorporates age and other factors to characterize life history using age-size SDMs with diffusion by introducing probability theory [8]. Their analysis showed that diffusion could change the generation time and $R_{0}$ even if the life histories were identical. They termed the diffusion “internal stochasticity”, which represents individual differences in life schedules, whereas the stochasticity on the population scale, such as environmental or demographic stochasticity, was designated as “external stochasticity”. The results of this paper raised the possibility that internal stochasticity leads to life histories based on Pianka’s classification. Moreover, Oizumi (2014) extended a previous work to unify a model of linear SDMs and stochastic control theory [9]. This theoretical model consists of two equations that both provide a more general characteristic equation based on Oizumi and Takada’s equation. These equations can simultaneously address the analysis of optimal life strategies and population dynamics under internal stochasticity. However, they do not account for density effects. Because the evolutionary diversity of life histories is viewed as the involvement of intraspecific competition at each population scale, Oizumi’s method should be extended to address *r*/*K*–selection theory.

To this end, this study establishes a more general framework containing both Oizumi’s methods and density effects. An appropriate fitness function is proposed that addresses both selections simultaneously. The function uses the Euler–Lotka equation in the absence of density effects and yields IRNI and $R_{0}$. Conversely, the function yields an equation where the density effects are satisfied at equilibrium. These properties of the function originate from Leon’s work based on age-SDMs [6]; however, the crucial difference is that the fitness function is generated by life histories, which are formulated using stochastic differential equations. Therefore, this study is able to treat not only age-specific SDMs but also arbitrary state-specific SDMs with diffusion. Applying our framework to specific models concerning semelparous species, we show that with internal stochasticity, we can reduce the carrying capacity. Furthermore, we present a specific example demonstrating that an adaptive risk hedge of internal stochasticity augments the carrying capacity and that it is ESS under *K*-selection.

## Framework for the mathematical methods

To address life histories in the presence of internal stochasticity and density effects, we outline our methodology and examine a general difference in adaptive life histories between *r*- and *K*-selection. The life histories of individuals are assumed to consist of four factors: growth, fertility, mortality, and interaction among individuals in our analysis. First, we define the models for these four factors. Second, we describe the population dynamics of the life history. Finally, the difference in adaptive strategies is discussed for both selections.

### General life history model

To link population-scale density effects to individual life histories, it is naturally thought that life histories should contain the density effects in the statistics related to the components, e.g., a mean size growth rate, the variance, etc. Moreover, individual life histories containing internal stochasticities are assumed to be diffusion processes of each state transition. Specifically, they include the migration of the population, size growth, and so on [8, 10–12]. Accordingly, we construct a general model of the life history influenced by diffusion processes with density effects. We define a set $A\subseteq R^{d}$ of all states. Suppose that *X*_*a*_ (*t*) ∈ *A* represents a *d*-dimensional state vector at age *a* and time *t* (e.g., size, weight, spatial position, etc.). The initial state is given as *X*_0_ (*t*) = *x*, which is independent of time *t*. Additionally, it is assumed that each state transition is regulated by an *l*-dimensional control vector *v* at the given age as per the individual life strategy [9]

$$\begin{array}{c} v=v_{a}\left(t\right):=\left(v_{1}\left(t,a\right),v_{2}\left(t,a\right),\cdots ,v_{l}\left(t,a\right)\right)\in V\subset R^{l}\;\left(l\geq 1\right), \end{array}$$

where V is a compact convex set of $R^{l}$ that denotes a set of all strategies. Given each state transition that is influenced by the *N*-dimensional internal stochasticities and parameterized by the *M*-dimensional density effect coefficient vector $\Gamma_{t,x}:=(\Gamma_{t,x}^{1},\Gamma_{t,x}^{2},\ldots ,\Gamma_{t,x}^{m},\ldots ,\Gamma_{t,x}^{M})$, which represents the magnitude of each density effect on the statistics of *X*_*a*_ (*t*) at time *t*, each diffusion process of states $X_{a}^{j}(t)$ is assumed to satisfy Ito’s stochastic differential equation as follows:

$$\begin{array}{c} \left\{\begin{array}{cc} dX_{a}^{j}\left(t\right)=g_{j}\left(X_{a}\left(t\right),v,\Gamma_{t,x}\right)da+\sum_{k=1}^{N}\sigma_{jk}\left(X_{a}\left(t\right),v,\Gamma_{t,x}\right)dB_{a}^{k}, & 1\leq j\leq d\\ X_{0}^{j}\left(t\right)=x^{j} \end{array}\right. \end{array}$$ (1)

where *x*^*j*^ (1 ≤ *j* ≤ *d*) represents an initial state of the *j*-th state, $X_{a}^{j}(t)$. The SDE is defined so that the difference in each state at an infinitesimally short time follows a Gaussian distribution. Because time *t* and age *a* are considered to have the same temporal development, the term “infinitesimally short time” is used here in the sense of direction of their characteristic line, i.e.,

$$\begin{array}{c} dX_{a}^{j}\left(t\right)=X_{a+\varepsilon }^{j}\left(t+\varepsilon \right)-X_{a}^{j}\left(t\right). \end{array}$$

This corresponds to the fact that the individual size growth and other transitions are different at each cohort. The first term *g*_*j*_ (⋅) on the right hand side in Eq (1) denotes the expectation of each state transition rate, and the second term denotes the fluctuation term and provides a covariance between another state provided by the Gaussian distribution, as follows:

$$\begin{array}{c} \begin{array}{cc} \lim_{\varepsilon ↓0}\frac{1}{\varepsilon }\text{Cov} & \left[\left|X_{a+\varepsilon }^{j}\left(t+\varepsilon \right)-X_{a}^{j}\left(t\right)\right|,\left|X_{a+\varepsilon }^{j^{\prime }}\left(t+\varepsilon \right)-X_{a}^{j^{\prime }}\left(t\right)\right|\right]\\ & =\sum_{k=1}^{N}\sigma_{jk}\left(X_{a}\left(t\right),v_{a},\Gamma_{t,x}\right)\sigma_{j^{\prime }k}\left(X_{a}\left(t\right),v_{a},\Gamma_{t,x}\right) \end{array} \end{array}$$

For the boundary value *x* ∈ ∂*A*, each state transition rate and the fluctuation term are assumed to be *g*_*j*_ (*x*, *v*, Γ_*t*,*x*_) = *σ*_*jk*_ (*x*, *v*, Γ_*t*,*x*_) = 0. This assumption means that several states attribute to the limited region. For instance, the domain of body-size is restricted to positive value. $B_{a}^{k}$ represents an element of *N*-dimensional Brownian motion generating internal stochasticity. Each element of the density effect coefficient vector Γ_*t*,*x*_ can be determined using the current density effects:

$$\begin{array}{c} \Gamma_{t,x}^{m}=\int_{0}^{\alpha }\int_{A}da\;dy\;\gamma^{m}\left(a,y\right)P_{t}\left(a,x\to y\right),\;\left(1\leq m\leq M\right) \end{array}$$ (2)

where *α* > 0, *γ*^*m*^ (*a*, *y*) ≥ 0 and $P_{t}(a,x\to y):[0,\infty )\times [0,\alpha )\times A\times A\to R_{+}:=[0,\infty )$ denote the maximum age, interaction coefficient of each generation and state, and population density of state *y* ∈ *A* at age *a* ∈ (0, *α*) in time *t* ∈ (0,∞), respectively. The diffusion process Eq (1) is complicated but includes the transition process in both *r*- and *K*-selection. When $0\in R_{+}^{M}$ is a zero vector, $\Gamma_{t,x}^{m}=\Gamma \approx 0$ represents *r*-selection. On the other hand, $\lim_{t↑\infty }\Gamma_{t,x}^{m}=\Gamma_{x}^{*}>0$ represents *K*-selection if and only if $\Gamma_{x}^{*}$ is unique. Then, *X*_*a*_ (*t*) becomes a diffusion process with respect to age in extreme environments where Γ_*t*,*x*_ is constant. Thus, Eq (1) accounts for the individual differences in the transition process that are characterized by each sample path and have different statistics for each population size. In addition, other components of the life histories are assumed to be functions with respect to *X*_*a*_ (*t*) and Γ_*t*,*x*_.

Let $F:A\times R_{+}^{M}\to R_{+}$ be a state-specific fertility, which depends on *y* and has integrability with respect to it for all $\Gamma \in R_{+}^{M}$ as follows:

$$\begin{array}{c} F\left(y,\Gamma_{t,x}\right)\geq 0. \end{array}$$ (3)

This definition of fertility *F* accounts for a wide class of breeding systems, such as a semelparous fertility with which the Dirichlet boundary conditions can be applied to population dynamics, as will be mentioned later on.

Let $\mu :R^{d}\times R^{l}\times R^{M}\to R_{+}$ be a mortality function depending on *y*, *v*, and Γ_*t*,*x*_ as follows:

$$\begin{array}{c} \mu_{0}\leq \mu \left(y,v,\Gamma_{t,x}\right)>0. \end{array}$$ (4)

The density effects on the fertility and the mortality are identical to the assumptions for the density-dependent age-structured models involved in the Allee effect and logistic effect [13]. We refer to Eqs (1), (3), and (4) as the life history.

### Dynamics at the population scale

In this subsection, we consider properties of the population dynamics derived from the components and statistics of the life history (Eqs (1), (3), and (4)). When *t* and *a* are fixed, the state transition of the population density *P*_*t*_ (*a*, *x* → *y*) at an infinitesimally short time *ε* follows an integral projection model:

$$\begin{array}{c} P_{t+\varepsilon }\left(a+\varepsilon ,x\to y\right)=\int_{A}d\xi \;K_{\varepsilon ,t}\left(\xi \to y\right)P_{t}\left(a,x\to \xi \right). \end{array}$$ (5)

*K*_*ε*, *t*_ (*ξ*→*y*) represents a projection function surviving and ranging from *ξ* to *y* at time interval *ε* and time *t*. The projection function is determined by Eqs (1) and (4). Let *n*_*t*_ (*x*) be the total neonatal density per unit time at time *t*, e.g., the total number of eggs and seeds, as well. The initial population density is written using *n*_*t*_ (*x*):

$$\begin{array}{c} P_{t}\left(0,x\to y\right)=n_{t}\left(x\right)\delta^{d}\left(x-y\right). \end{array}$$ (6)

*δ*^*d*^ (*x* − *y*) is the *d*-dimensional Dirac’s delta

$$\begin{array}{c} \delta^{d}\left(x-y\right):=\prod_{j=1}^{d}\delta \left(x^{j}-y^{j}\right). \end{array}$$

In addition, the neonatal dynamics follow

$$\begin{array}{c} n_{t}\left(x\right)=\int_{0}^{\alpha }\int_{A}dady\;F\left(y,\Gamma_{t,x}\right)P_{t}\left(0,x\to y\right). \end{array}$$ (7)

Using the components of the life history Eqs (1), (3), and (4), we derive a non-linear partial differential equation from Eq (5) (Cf. Text A in S1 File) as follows:

$$\begin{array}{c} \left\{\begin{array}{cc} & \left[\frac{\partial }{\partial t}+\frac{\partial }{\partial a}\right]P_{t}\left(a,x\to y\right)=-H_{y}^{v}\left(\Gamma_{t,x}\right)P_{t}\left(a,x\to y\right)\\ & H_{y}^{v}\left(\Gamma_{t,x}\right):=\sum_{j=1}^{d}\frac{\partial }{\partial y^{j}}g_{j}\left(y,v,\Gamma_{t,x}\right)-\frac{1}{2}\sum_{j,j^{\prime }=1}^{d}\frac{\partial^{2}}{\partial y^{j}\partial y^{j^{\prime }}}c_{jj^{\prime }}\left(y,v,\Gamma_{t,x}\right)+\mu \left(y,v,\Gamma_{t,x}\right)\\ & P_{0}\left(a,x\to y\right)=P\left(a,x,y\right), \end{array}\right. \end{array}$$ (8)

where *c*_*jj*′_ (*y*, *v*, Γ_*t*,*x*_) denote

$$\begin{array}{c} c_{jj^{\prime }}\left(y,v,\Gamma_{t,x}\right):=\sum_{k=1}^{N}\sigma_{jk}\left(y,v,\Gamma_{t,x}\right)\sigma_{j^{\prime }k}\left(y,v,\Gamma_{t,x}\right). \end{array}$$

$P(a,x,y):[0,\alpha )\times A\times A\to R_{+}$ is the initial population density. It is then assumed that all individuals have the same initial state *x*. The differential operator $H_{y}^{v}(\Gamma_{t,x})$ is referred to as the Fokker–Planck Hamiltonian operator, which provides the state transition with diffusion. To analyze the adaptive strategies in *r*/*K*-selection, it is important to determine the asymptotic behavior of the population dynamics at the two equilibria representing each selection. Hence, we focus on the population sizes satisfying

$$\begin{array}{c} \left[\frac{\partial }{\partial t}+\frac{\partial }{\partial a}\right]P_{t}^{0}\left(a,x\to y\right)=-H_{y}^{v}\left(0\right)P_{t}^{0}\left(a,x\to y\right), \end{array}$$ (9)

and

$$\begin{array}{c} \frac{\partial }{\partial a}P^{*}\left(a,x\to y\right)=-H_{y}^{v}\left(\Gamma_{x}^{*}\right)P^{*}\left(a,x\to y\right), \end{array}$$ (10)

where $0\in R_{+}^{M}$ and $\Gamma_{x}^{*}\in R_{+}^{M}$ denote the zero vector and density effect coefficients for a non-trivial equilibrium, respectively. Eq (9) provides the asymptotic behavior of the population size, which is sufficiently small so that the density effect can be neglected: ($\Gamma_{x}^{*}\approx 0$), i.e., it represents *r*-selection. On the other hand, Eq (10) implies *K*-selection, where the size reaches a non-trivial and stable equilibrium. Both conditions can be rewritten using a characteristic function

$$\begin{array}{c} \psi_{\lambda }^{v}\left(x,\Gamma \right):=\int_{0}^{\alpha }da\;\exp\left\{-\lambda a\right\}E_{x}\left[F\left(X_{a},\Gamma \right)\exp\left\{-\int_{0}^{a}d\tau \;\mu \left(X_{\tau },v_{\tau },\Gamma \right)\right\}\right]. \end{array}$$ (11)

where λ∈R and $\Gamma \in R_{+}^{M}$ are constant and constant vector, respectively. The expectation $E_{x}[\cdot ]$ is given by

$$\begin{array}{c} E_{x}\left[F\left(X_{a},\Gamma \right)\exp\left\{-\int_{0}^{a}d\tau \;\mu \left(X_{\tau },v_{\tau },\Gamma \right)\right\}\right]:=\int_{A}dy\;F\left(y,\Gamma \right)K_{a}\left(x\to y\right). \end{array}$$ (12)

The projection function *K*_*a*_ (*x* → *y*), then, is the solution of

$$\begin{array}{c} \left\{\begin{array}{cc} & \left[\frac{\partial }{\partial a}+H_{y}^{v}\left(\Gamma \right)\right]K_{a}\left(x\to y\right)=0\\ & K_{0}\left(x\to y\right)=\delta^{d}\left(x-y\right), \end{array}\right. \end{array}$$ (13)

which represents the density surviving and growing from *x* to *y* at age *a* under Eq (1), providing the statistical rule. The IRNI of Eq (9) is provided by λ* satisfying

$$\begin{array}{c} \psi_{\lambda }^{v}\left(x,\Gamma \right){|}_{\lambda =\lambda^{*},\Gamma =0}=1, \end{array}$$ (14)

[8, 9], whereas, $\Gamma_{x}^{*}$ in Eq (10) satisfies

$$\begin{array}{c} \psi_{\lambda }^{v}\left(x,\Gamma \right){|}_{\lambda =0,\Gamma =\Gamma_{x}^{*}}=1. \end{array}$$ (15)

The former implies the characteristic equation of Eq (9) (also called the Euler–Lotka equation), and the latter represents that $R_{0}$ is equal to one at equilibrium. Thus, Eq (11) can address various steady states of Eq (8) with different values of Γ. In other words, Eq (11) represents the reproductive contributions of all cohorts to the population dynamics for each population size.

Further, the logarithm of the characteristic function Eq (11) is composed of the cumulant generating function of the breeding age a*, which contributes to reproduction, such that

$$\begin{array}{c} \begin{array}{c} \text{ln}\psi_{\lambda }^{v}\left(x,\Gamma \right)=\sum_{k=0}^{\infty }\frac{{\left(-\lambda \right)}^{k}}{k!}{\langle a^{*}\rangle }_{x,\Gamma }^{\left(k\right)}, \end{array} \end{array}$$ (16)

where ${\langle a^{*}\rangle }_{x,\Gamma }^{\left(k\right)}$ represents the *k*-th cumulant [8]. These statistics are provided by a breeding age distribution parameterized by Γ

$$\begin{array}{c} A\left(a,\Gamma \right):=\frac{1}{\psi_{0}^{v}\left(x,\Gamma \right)}E_{x}\left[F\left(X_{a},\Gamma \right)\exp\left\{-\int_{0}^{a}d\tau \;\mu \left(X_{\tau },v_{\tau },\Gamma \right)\right\}\right]. \end{array}$$ (17)

This distribution is important for considering the effect of *r*/*K*–selection on the breeding system. Therefore, Eq (11) can be used as a common criterion in both selections and plays a pivotal role in this study.

The life histories of *r*/*K*-selection are based on the assumption that Eq (1) has a logistic type of behavior; (i) Eq (8) has a unique equilibrium population density except at zero (P* (*a*, *x* → *y*) > 0), and (ii) the vector is stable for any v∈V when the other (P* (*a*, *x* → *y*) = 0) is unstable, as follows:

$$\begin{array}{c} \psi_{\lambda }^{v}\left(x,\Gamma \right){|}_{\lambda =0,\Gamma =0}>1, \end{array}$$ (18)

Under conditions (i) and (ii), the carrying capacity can be derived from the density effect at equilibrium:

$$\begin{array}{c} \Gamma_{x}^{m*}=\int_{0}^{\alpha }\int_{A}dady\;\gamma^{m}\left(a,y\right)P^{*}\left(a,x\to y\right). \end{array}$$ (19)

It is known that the equilibrium population can be decomposed into the total neonatal density n*(*x*) and the projection function $K_{a}^{*}(x\to y)$, such that

$$\begin{array}{c} P^{*}\left(a,x\to y\right)=n^{*}\left(x\right)K_{a}^{*}\left(x\to y\right), \end{array}$$ (20)

[9]. Then, the projection function represents a density at which individuals survive and reach *y* from *x* at age *a* under the density effect $\Gamma_{x}^{*}$. The projection function is composed of

$$\begin{array}{c} \psi_{0}^{v}\left(x,\Gamma_{x}^{*}\right)=\int_{0}^{\alpha }\int_{A}dady\;F\left(y,\Gamma_{x}^{*}\right)K_{a}^{*}\left(x\to y\right), \end{array}$$ (21)

and hence, we have

$$\begin{array}{c} \Gamma_{x}^{m*}=n^{*}\left(x\right)\int_{0}^{\alpha }da\;E_{x}^{*}\left[\gamma^{m}\left(a,X_{a}\right)\exp\left\{-\int_{0}^{a}d\tau \;\mu \left(X_{\tau },v_{\tau },\Gamma_{x}^{*}\right)\right\}\right], \end{array}$$ (22)

where $E_{x}^{*}$ denotes the expectation of the projection function under the non-trivial equilibrium, $K_{a}^{*}(x\to y)$. From this, we obtain

$$\begin{array}{c} P^{*}\left(a,x\to y\right)=\frac{\Gamma_{x}^{m*}K_{a}^{*}\left(x\to y\right)}{\int_{0}^{\alpha }da\;E_{x}^{*}\left[\gamma^{m}\left(a,X_{a}\right)\exp\left\{-\int_{0}^{a}d\tau \;\mu \left(X_{\tau },v_{\tau },\Gamma_{x}^{*}\right)\right\}\right]}, \end{array}$$ (23)

for all *m*. If this equilibrium is stable (the condition referred to Text B in S1 File), the total population size $N^{*}$ is equivalent to the carrying capacity and is

$$\begin{array}{c} \begin{array}{cc} N^{*}: & =\int_{0}^{\alpha }\int_{A}dady\;P^{*}\left(a,x\to y\right)\\ & =\frac{\Gamma_{x}^{m*}\int_{0}^{\alpha }da\;E_{x}^{*}\left[\exp\left\{-\int_{0}^{a}d\tau \;\mu \left(X_{\tau },v_{\tau },\Gamma_{x}^{*}\right)\right\}\right]}{\int_{0}^{\alpha }da\;E_{x}^{*}\left[\gamma^{m}\left(a,X_{a}\right)\exp\left\{-\int_{0}^{a}d\tau \;\mu \left(X_{\tau },v_{\tau },\Gamma_{x}^{*}\right)\right\}\right]}\;\text{for}\;\text{all}\;m. \end{array} \end{array}$$ (24)

Conventional *K*-selection theory argues that adaptive strategies maximize $N^{*}$. Taking into account the influence of the population structure, the argument should be reconsidered at the individual scale. For instance, when two different species with the same carrying capacity invade identical habitats, conventional *K*-selection theory cannot assess whether either of the two life histories is adaptive. To address this sort of question, we present an answer to the one mentioned above using an extension of Oizumi’s method to incorporate nonlinear systems in the next section.

### Adaptive life histories

At the beginning of the analysis, we introduce an important theorem related to Eq (11):

**Theorem 1**. *Let* $\tilde{v}$ *be*

$$\begin{array}{c} \tilde{v}\in V,\;s.t.\;\lambda^{*,\tilde{v}}=\underset{v}{\sup}\lambda^{*,v}, \end{array}$$

*and* $\psi_{\lambda }^{v}(x,\Gamma )$ *be given by* Eq (11) *and* $\psi_{\lambda^{*,v}}^{v}(x,\Gamma )=1$, *such that*

$$\begin{array}{c} \psi_{\lambda }^{v}\left(x,\Gamma \right)=\int_{0}^{\alpha }da\;w_{\lambda ,a}\left(x,\Gamma \right). \end{array}$$

*Define* $\tilde{\lambda }$ *by* $\tilde{\lambda }:=\lambda^{*,\tilde{v}}$. *Then, we have*

$$\begin{array}{c} \psi_{\tilde{\lambda }}^{v}\left(x,\Gamma \right)\leq \psi_{\tilde{\lambda }}^{\tilde{v}}\left(x,\Gamma \right)=1\Leftrightarrow \lambda^{*,v}\leq \lambda^{*,\tilde{v}}. \end{array}$$

This theorem describes that a strategy $\tilde{v}$ for maximizing Eq (11) is equivalent to maximizing λ*. This theorem is easily verified because of the monotonicity of Eq (11) with respect to *λ*. Theorem 1 provides that $\tilde{v}$ is defined as the adaptive strategy at Γ. Accordingly, we adopt Eq (11) as the fitness function in this paper. To find $\tilde{v}$, the maximized characteristic function is to be determined. Hence, we introduce Oizumi’s equations parameterized by Γ such that

$$\begin{array}{c} \left\{\begin{array}{cc} & \frac{\partial }{\partial a}{\tilde{w}}_{\lambda ,a}\left(x,\Gamma \right)-\underset{v\in V}{\inf}\left\{\left[{\bar{H}}_{x}^{v}\left(\Gamma \right)+\lambda \right]{\tilde{w}}_{\lambda ,a}\left(x,\Gamma \right)\right\}=0\\ & {\tilde{w}}_{\lambda ,\alpha }\left(x,\Gamma \right)=F\left(x,\Gamma \right)\\ & {\bar{H}}_{x}^{v}\left(\Gamma \right):=-\sum_{j=1}^{d}g_{j}\left(x,v,\Gamma \right)\frac{\partial }{\partial x^{j}}-\frac{1}{2}\sum_{j,j^{\prime }=1}^{d}c_{jj^{\prime }}\left(x,v,\Gamma \right)\frac{\partial^{2}}{\partial x^{j}\partial x^{j^{\prime }}}+\mu \left(x,v,\Gamma \right), \end{array}\right. \end{array}$$ (25)

$$\begin{array}{c} {\tilde{\psi }}_{\lambda }\left(x,\Gamma \right)=\int_{0}^{\alpha }da\;{\tilde{w}}_{\lambda ,a}\left(x,\Gamma \right)=1, \end{array}$$ (26)

Eq (25) is one of the Hamilton–Jacobi–Bellman (HJB) equations in Control Theory [14], the solution of which, ${\tilde{w}}_{\lambda ,a}(x,\Gamma )$, is the following function described using the age in descending order (*a* → *α* − *a*);

$$\begin{array}{c} \begin{array}{cc} & {\tilde{w}}_{\lambda ,a}\left(x,\Gamma \right)\\ & :=\underset{v\in V}{\sup}\left\{\exp\left\{-\lambda \left(\alpha -a\right)\right\}E_{x}\left[F\left(X_{\alpha -a},\Gamma \right)\exp\left\{-\int_{0}^{\alpha -a}d\tau \;\mu \left(X_{\tau },v_{\tau },\Gamma \right)\right\}\right]\right\}. \end{array} \end{array}$$ (27)

The reason behind the variable change in age originated from Bellman’s principle with regard to derivation of the HJB equation (Cf. Text C in S1 File and [9]). ${\bar{H}}_{x}^{v}(\Gamma )$ represents the formal adjoint operator of $H_{y}^{v}(\Gamma )$ in terms of the variable *x* in Eq (8). Then, Eq (27) represents the maximized reproductive contribution of cohort at age *a*, which includes a maximized characteristic function, such that

$$\begin{array}{c} {\tilde{\psi }}_{\lambda }\left(x,\Gamma \right):=\int_{0}^{\alpha }da\;{\tilde{w}}_{\lambda ,a}\left(x,\Gamma \right). \end{array}$$ (28)

Eq (26) is a characteristic equation of the adaptive species.

Eqs (25) and (26) provide a dominant characteristic root $\tilde{\lambda }$ and the adaptive strategy $\tilde{v}$ as functions of Γ:

$$\begin{array}{c} \tilde{\lambda }=\tilde{\lambda }\left(x,\Gamma \right)\;\tilde{v}=\tilde{v}\left(\alpha -a,X_{a},\Gamma \right), \end{array}$$ (29)

where the strategy is expressed in terms of the original age *a*. We then add a new assumption as follows: (iii) Eq (29) exists and is unique. Substituting Γ = **0** into Eq (29), $\tilde{\lambda }(x,0)$ represents the IRNI of the adaptive species and $\tilde{v}(\Gamma =0)$ denotes the adaptive strategy in the absence of the density effect, which is referred to as the *r*-strategy ${\tilde{v}}_{r}$. In contrast, we show that in the presence of the density effect, $\tilde{\Gamma }$ satisfying $\tilde{\lambda }=0$ provides the adaptive strategy in *K*-selection (*K*-strategy:${\tilde{v}}_{K}=\tilde{v}(\Gamma =\tilde{\Gamma })$).

### *K*-strategy

Substituting $\Gamma =\tilde{\Gamma }$ into Eq (26) gives

$$\begin{array}{c} {\tilde{\psi }}_{0}\left(x,\tilde{\Gamma }\right)=1. \end{array}$$ (30)

Theorem 1 indicates that the adaptive species under the density effects $\tilde{\Gamma }$ has the maximum IRNI $\tilde{\lambda }$ equal to zero: the strategy $\tilde{v}(\Gamma =\tilde{\Gamma })$ is adaptive in terms of the maximum density effect. Suppose that individuals with a strategy *v* reach an equilibrium given by Γ*. If for Γ*, $\tilde{\lambda }(x,\Gamma^{*})>0$ in Eq (29), the population can be invaded because there exists a strategy that allows individuals to bear more offspring with the given population size. No strategy, however, can invade $\tilde{v}(\Gamma =\tilde{\Gamma })$ at equilibrium because

$$\begin{array}{c} \psi_{0}^{v}\left(x,\tilde{\Gamma }\right)\leq {\tilde{\psi }}_{0}\left(x,\tilde{\Gamma }\right)=1, \end{array}$$ (31)

for all *v*. Accordingly, $\tilde{v}(\Gamma =\tilde{\Gamma })$ is consistent with the definition of ESS; hence, we call this strategy *K*-strategy ${\tilde{v}}_{K}$. Eq (31), then, has a comparable meaning to the evolutionary invasion analysis in AD. Focusing on what *K*-strategy modifies on the population scale, the possibility is raised that ${\tilde{v}}_{K}$ generates complicated state structures of the population depending on each interaction coefficient *γ*^*m*^ (*a*, *y*). It is significantly different from traditional *K*-selection theory because ${\tilde{v}}_{K}$ does not maximize the carrying capacity. This difference is accounted for by considering the structure of the intraspecific interaction as the density effect. If and only if the *m*′-th interaction coefficient in Eq (2) is constant, $\gamma^{m^{\prime }}(a,y)\equiv \gamma_{0}^{m^{\prime }}$, $\tilde{\Gamma }$ is equivalent to the maximum carrying capacity as follows:

$$\begin{array}{c} N^{*}=\frac{\Gamma_{x}^{m^{\prime }*}}{\gamma_{0}^{m^{\prime }}}\leq \frac{{\tilde{\Gamma }}_{x}^{m^{\prime }}}{\gamma_{0}^{m^{\prime }}}, \end{array}$$ (32)

This may not be necessarily true otherwise.

Taken together, ${\tilde{v}}_{K}$ contains classical *K*-selection theory as a particular case. Eqs (25) and (26) can possibly be applied to OLSP influenced by various effects of intraspecific competition and internal stochasticity. In order to show the utility of both equations, in the next section, we illustrate that there exists a specific model matching our framework and examine how both effects influence the life history using the model.

### Specific example

This section addresses a specific model following the assumptions (i)-(iii) to examine how the density effect and internal stochasticity influence the adaptive strategy. As a mathematical example, the model which we would handle is suggestive on the examination because it has the exact solution and several solvable indices. By use of the example, we then focus on these effects for alternations of generations. According to Pianka’s classification mentioned in the Introduction [3], *r*-strategy has a shorter generation time than other strategies. To examine this, we demonstrate in the next subsection that internal stochasticity affects the breeding age density (see Eq (17)) and the carrying capacity. Second, we analyze the evolution of adaptive life history by using two-resource utilization models following the former results. Then, the adaptive strategy of the model is analyzed in terms of the statistics of the breeding age compared with Pianka’s table.

#### Resource acquisition competition model

The role of internal stochasticity at the population scale should be clarified before analyzing adaptive control. Accordingly, we start by analyzing the effect of stochasticity on the life history without control parameters. Suppose that the size of the individuals $X_{a}^{1}\in A\subset R_{+}$ grows in accordance with a geometric Brownian motion and that the average size growth rate is reduced by intraspecific competition for foraging Γ_*t*_ as follows:

$$\begin{array}{c} \left\{\begin{array}{cc} dX_{a}^{1} & =\left[b_{1}-\Gamma \right]X_{a}^{1}da+\sigma_{1}X_{a}^{1}dB_{a}^{1}\\ X_{0}^{1} & =x, \end{array}\right. \end{array}$$ (33)

where $b_{1},\Gamma ,\sigma_{1}\in R_{+}$. If Γ_*t*_ does not exist, the size of the individuals grows exponentially with fluctuation. We call this model the resource acquisition competition model.

The breeding system is assumed to be semelparity. Let x* > *x* be the mature size, and let the breeding age a* be defined as the first age when a size reaches the mature size as follows:

$$\begin{array}{c} X_{a^{*}}=x^{*},\;a^{*}:=\inf\left\{a\in \left(0,\infty \right]|X_{a}\geq x^{*}\right\}. \end{array}$$ (34)

The breeding age is, therefore, not unique but is represented using a statistic because of the noise. We refer to the age as the mature age in this model. Therefore, the mature age is a statistic because of the noise. The size-specific fertility *F*_*S*_ (*y*), then, is defined by

$$\begin{array}{c} F_{S}\left(y\right):=\left\{\begin{array}{cc} \phi \left(y\right) & y=x^{*}\\ 0 & y\neq x^{*} \end{array}\right. \end{array}$$ (35)

where the fertility rate function $\phi (y):R_{+}\to R_{+}$ denotes the fecundity per individual, which satisfies *ϕ*(x*) > 1 at the mature size.

The mortality in Eq (4) is assumed to be

$$\begin{array}{c} \mu \left(y,v\right)=\left\{\begin{array}{cc} \mu_{0} & a\leq a^{*}\\ \infty & a>a^{*} \end{array}\right. \end{array}$$ (36)

where *μ*_0_ > 0, which does not have control parameters. Because semelparous species die upon reproduction, we can write the survivorship as

$$\begin{array}{c} S_{S}\left(a\right):=\left\{\begin{array}{cc} \exp\left\{-\mu_{0}a\right\} & a\leq a^{*}\\ 0 & a>a^{*} \end{array}\right.. \end{array}$$ (37)

The interaction coefficient *γ* (*a*, *y*) is assumed to be constant as follows;

$$\begin{array}{c} \gamma \left(a,y\right)\equiv \gamma_{0}. \end{array}$$ (38)

Eq (38) seems to be oversimplified, but it is not important for the adaptive strategy because the interaction coefficients of our framework are only related to the stability and population size at equilibrium. These assumptions of semelparous life history are reasonable for comparing differences between the absence and the presence of the density effect because each individual bears an identical number of offspring. Accordingly, the density effect is only involved in the statistics of the mature age; in other words, it is sufficient to pay attention to the effect on generation time and the size of Γ*.

## Results

### Statistics of the life history in *r*/*K*–selection

The characteristic function Eq (11) can be derived from Eqs (33), (35), and (37). Because the mature age a* attributes lie between zero and infinity, i.e., a* ∈ (0,∞] (a* = ∞ means that individuals do not reach the mature size), the maximum age *α* is equal to infinity. Then, it is known from [8] that the characteristic function *ψ*_*Sλ*_ (*x*, Γ) is a solution of

$$\begin{array}{c} \begin{array}{cc} & -\left[{\bar{H}}_{x}\left(\Gamma \right)+\lambda \right]\psi_{S\lambda }\left(x,\Gamma \right)+F_{S}\left(x\right)=0\\ & {\bar{H}}_{x}\left(\Gamma \right)=-\left(b_{1}-\Gamma \right)x\frac{d}{dx}-\frac{1}{2}\sigma_{1}^{2}x^{2}\frac{d^{2}}{dx^{2}}+\mu_{0}. \end{array} \end{array}$$ (39)

The equation above has the following solution (the derivation is provided on pp.87-88 in [8]),

$$\begin{array}{c} \begin{array}{cc} \psi_{S\lambda }\left(x,\Gamma \right) & =E_{x}\left[\exp\left\{-\left(\lambda +\mu_{0}\right)a^{*}\right\}\right]\phi \left(x^{*}\right)={\left(\frac{x}{x^{*}}\right)}^{\rho_{\lambda }\left(\Gamma \right)}\phi \left(x^{*}\right)\\ \rho_{\lambda }\left(\Gamma \right): & =\frac{1}{2}\left(1-\frac{2\left(b_{1}-\Gamma \right)}{\sigma_{1}^{2}}\right)+\frac{1}{2}\sqrt{{\left(1-\frac{2\left(b_{1}-\Gamma \right)}{\sigma_{1}^{2}}\right)}^{2}+\frac{8\mu_{0}}{\sigma_{1}^{2}}+\frac{8\lambda }{\sigma_{1}^{2}}}. \end{array} \end{array}$$ (40)

Eqs (14), (15), and (24) provide the IRNI and total population at equilibrium that are

$$\begin{array}{c} \lambda^{*}=\left[b_{1}-\frac{\sigma_{1}^{2}}{2}\left(1-\rho^{*}\right)\right]\rho^{*}-\mu_{0}, \end{array}$$ (41)

and

$$\begin{array}{c} N^{*}=\frac{\Gamma_{x}^{*}}{\gamma_{0}}=\frac{1}{\gamma_{0}}\left[b_{1}-\frac{\sigma_{1}^{2}}{2}\left(1-\rho^{*}\right)-\frac{\mu_{0}}{\rho^{*}}\right], \end{array}$$ (42)

respectively. ρ* denotes

$$\begin{array}{c} \rho^{*}:=\frac{\ln\phi \left(x^{*}\right)}{\ln\frac{x^{*}}{x}}. \end{array}$$ (43)

**Fig 1:**
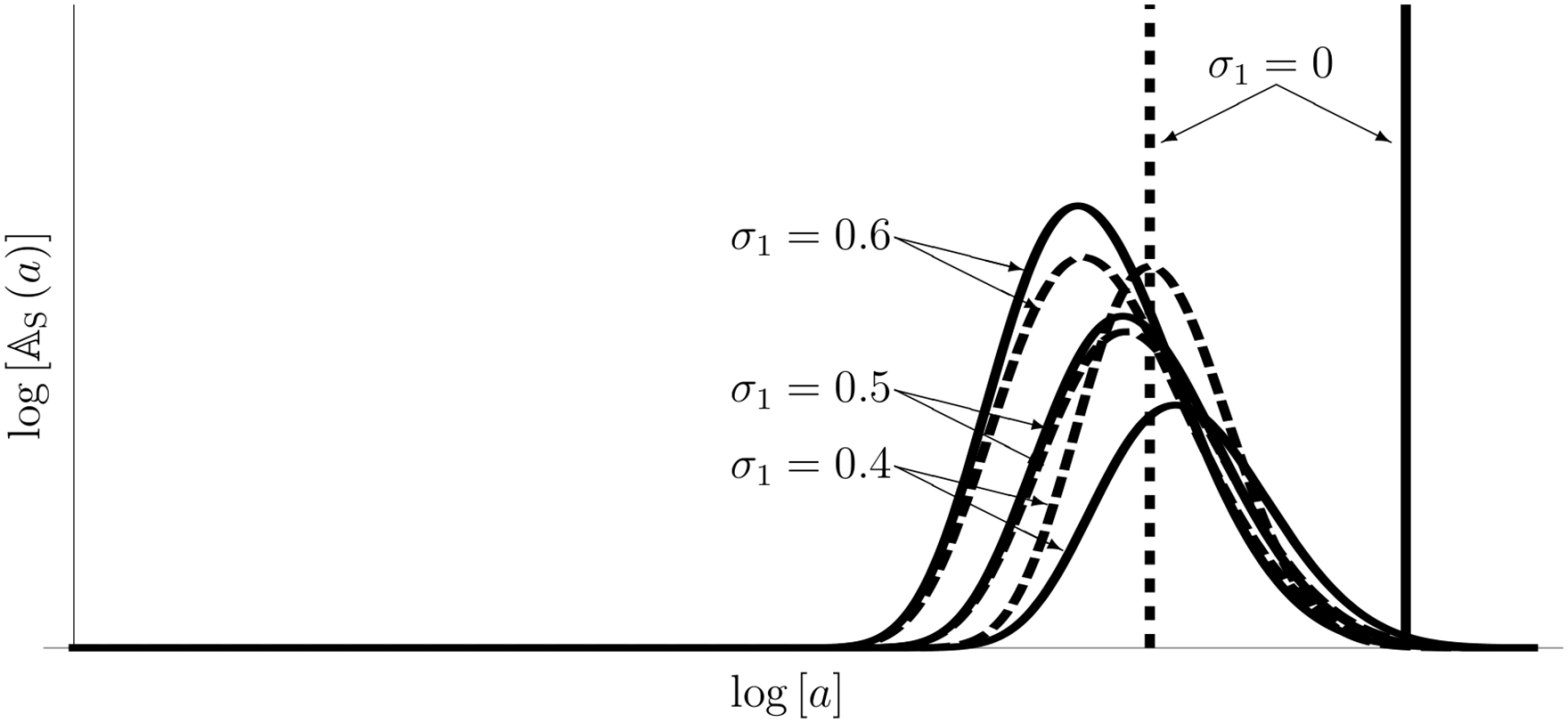
Sensitivity of the mature age density to the internal stochasticity. This figure illustrates the effect of the magnitude of noise on the mature age density under both *r*- and *K*-selection. The horizontal axis denotes the logarithmic scale of the age, and the vertical axis denotes the height of the mature age density function on the logarithmic scale. The solid line and the dashed line represent the mature age density functions under *K*-selection and *r*-selection, respectively. Both mature ages are biased to precocity depending on the magnitude of the noise; furthermore, we see that *r*-selection does not always accelerate maturity under internal stochasticity. The parameters of this simulation are *b*_1_ = 0.15, *σ*_1_ = 0.4,0.5,0.6, *x* = 0.01, x* = 1.5, *ϕ*(x*) = 10, and *μ*_0_ = 0.01.

If the IRNI is positive, Eq (42) becomes the carrying capacity, i.e., it is unique and stable (cf. Text D in S1 File). To examine the relationship between the mature age structure and internal stochasticity, we derive Eq (17) from Eq (40) (cf. Text E in S1 File). Fig 1 illustrates the effect of internal stochasticity on the mature age density, implying that the stochasticity promotes maturity in both *r*- and *K*-selection. According to the previous paper [8], the bias of the density is due to low survivorship of late-mature individuals.

Focusing on the effect of internal stochasticity on the IRNI and the carrying capacity, it was shown in the previous paper that the sensitivity of the IRNI with respect to the magnitude of the stochasticity *σ*_1_ is determined by ρ* being greater or less than one (cf. [8]). If ρ* is greater than one, the sensitivity is positive; otherwise, it is zero or negative. Similarly, the carrying capacity has an identical condition:

$$\begin{array}{c} \frac{\partial }{\partial \sigma_{1}^{2}}N^{*}=-\frac{1}{2\gamma_{0}}\left(1-\rho^{*}\right). \end{array}$$ (44)

Furthermore, because the sensitivities of the IRNI and $N^{*}$ with respect to ρ* are always positive in the persistent domain (λ* > 0 i.e., ρ* > *ρ*_0_(0)), the adaptive mature size $\tilde{x}$ is obtained using the chain rule in calculus as follows:

$$\begin{array}{c} \frac{\partial \lambda^{*}}{\partial \rho^{*}}\frac{\partial \rho^{*}}{\partial x^{*}}{|}_{x^{*}=\tilde{x}}=\frac{\partial N^{*}}{\partial \rho^{*}}\frac{\partial \rho^{*}}{\partial x^{*}}{|}_{x^{*}=\tilde{x}}=0. \end{array}$$ (45)

That is,

$$\begin{array}{c} \tilde{\rho }=\frac{\ln\phi \left(\tilde{x}\right)}{\ln\frac{\tilde{x}}{x}}=\underset{x^{*}\in A}{\sup}\frac{\ln\phi \left(x^{*}\right)}{\ln\frac{x^{*}}{x}}. \end{array}$$ (46)

$\tilde{x}$ is, thus, ESS in both selections, which means that nature selects a mature size maximizing ρ*. Because ρ* plays a key role in the risk hedging of the life history, we term it “Efficiency Exponent of Semelparity” (EES). Maximizing EES evokes two different interpretations; one is that the size maximizes the number of the individual’s own offspring in the numerator, and the other reduces the proportion of the initial size to the mature size in the denominator. The latter accelerates alternations of generations. If the EES is equal to one, the influence of internal stochasticity disappears, which is interpreted as the remaining valance between the length of the life span and basic reproductive number. This suggests that species with an EES of one seem to follow the deterministic rule *σ*_1_ = 0.

Internal stochasticity becomes a negative risk for species with EES less than one ρ* < 1 because of increasing the risk of extinction by reduction of the carrying capacity and delaying recovery of the population from large disturbances. An optimal risk hedging is required for the life history. We consider the optimal risk hedging of resource utilization as an application of Eqs (25) and (26) in the next section.

### Density-dependent two-resource utilization model

Based on Eqs (33), (35), (36), and (38), we consider a species utilizing different resources (we call them *R*_1_ and *R*_2_). The size of a specialist utilizing *R*_1_ is the following growth rate

$$\begin{array}{c} \left\{\begin{array}{cc} dX_{a}^{1} & =\left[b_{1}-\kappa \Gamma \right]X_{a}^{1}da+\sigma_{1}X_{a}^{1}dB_{a}^{1}\\ X_{0}^{1} & =x, \end{array}\right., \end{array}$$ (47)

and that of utilizing the other resource, *R*_2_, is

$$\begin{array}{c} \left\{\begin{array}{cc} dX_{a}^{2} & =\left[b_{2}-\left(1-\kappa \right)\Gamma \right]X_{a}^{2}da+\sigma_{2}X_{a}^{2}dB_{a}^{2}\\ X_{0}^{2} & =x. \end{array}\right. \end{array}$$ (48)

$B_{a}^{1}$ and $B_{a}^{2}$ are Brownian motions that are independent of each other. We then assume that with $b_{1}\in R_{+}>b_{2}\in R$ (*b*_2_ could be negative), *σ*_1_ > *σ*_2_ ≥ 0, i.e., if Γ_*t*_ ≈ 0, choosing *R*_1_ implies a higher risk and expectation of growth rate than choosing *R*_2_. Conversely, choosing *R*_2_ under the same conditions confers another risk that individuals have a lower survivorship until they reach a mature age than choosing *R*_1_ because of the slower growth rate, on average. A constant *κ* ∈ (0,1) denotes the ratio of strength in intraspecific competition, which alters the statistically significant difference between these resources. Therefore, individuals should find their optimal risk hedging, ${\tilde{v}}_{a}\in [0,1]$, in accordance with each population size under the following growth rate:

$$\begin{array}{c} \left\{\begin{array}{cc} dX_{a} & =\left[\theta_{1}\left(1-v_{a}\right)+\theta_{2}v_{a}\right]X_{a}da+\left[\sigma_{1}\left(1-v_{a}\right)dB_{a}^{1}+\sigma_{2}v_{a}dB_{a}^{2}\right]X_{a}\\ X_{0} & =x, \end{array}\right. \end{array}$$ (49)

where

$$\begin{array}{c} \theta_{1}:=b_{1}-\kappa \Gamma ,\;\theta_{2}:=b_{2}-\left(1-\kappa \right)\Gamma . \end{array}$$ (50)

Eq (49) is referred as to the density-dependent two-resource utilization model, satisfying the conditions (i-iii) when EES is less than one (the reason for the model satisfying the condition (iii) is mentioned in a later section). This model generates the following adjoint Fokker–Planck Hamiltonian,

$$\begin{array}{c} \begin{array}{cc} & {\bar{H}}_{x}^{v}\left(\Gamma \right)=\\ & -\left(\theta_{1}\left(1-v\right)+\theta_{2}v\right)x\frac{d}{dx}-\frac{1}{2}\left(\sigma_{1}^{2}{\left(1-v\right)}^{2}+\sigma_{2}^{2}v^{2}\right)x^{2}\frac{d^{2}}{dx^{2}}+\mu_{0}. \end{array} \end{array}$$ (51)

### Adaptive utilization and density effect

Because our model is *α* = ∞, the characteristic function of the adaptive species satisfies

$$\begin{array}{c} -\underset{v\in V}{\inf}\left\{\left[{\bar{H}}_{x}^{v}\left(\Gamma \right)+\lambda \right]{\tilde{\psi }}_{\lambda }\left(x,\Gamma \right)\right\}+F\left(x,\Gamma \right)=0, \end{array}$$ (52)

(Cf. Text C in S1 File).

**Fig 2:**
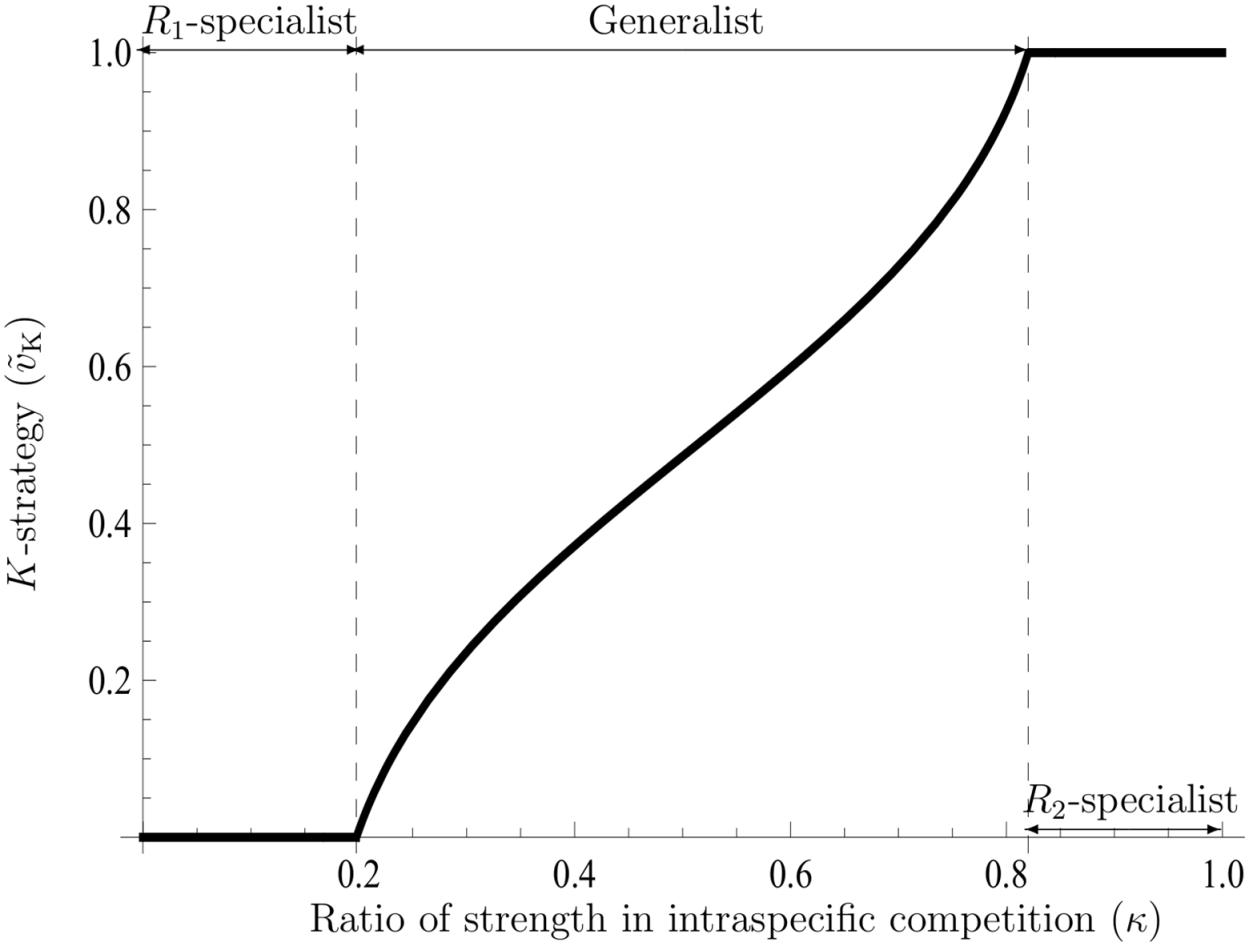
The *K*-strategy of the two-resource utilization model. This figure shows the shape of adaptive utilization under *K*-selection with respect to the ratio of the strength in the intraspecific competition *κ*. As the ratio increases, the adaptive strategy shifts to *R*_1_-specialists, generalists, and *R*_2_-specialists in turn. It is known that *R*_2_-specialists do not evolve in the absence of density effects [9]. The parameters are *b*_1_ = 0.15, *b*_2_ = 0.05, *σ*_1_ = 0.6, *σ*_2_ = 0.02, *x* = 0.01, x* = 1.5, *ϕ*(x*) = 10, and *μ*_0_ = 0.01.

**Fig 3:**
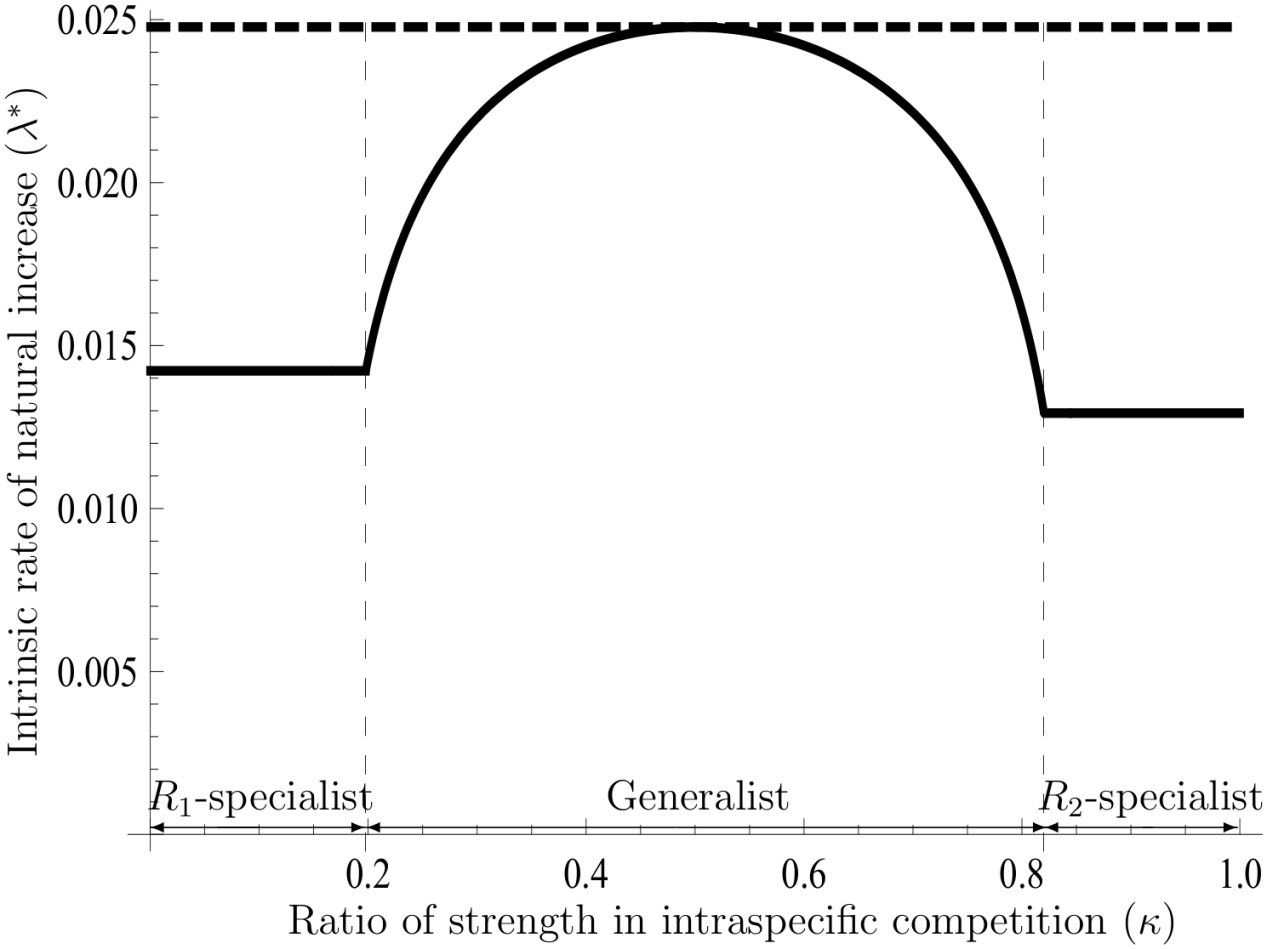
Differences in the intrinsic rate of the natural increase between both strategies. The figure illustrates the values of IRNI in the *r*- and *K*-strategies. The horizontal axis denotes the ratio of strength in the intraspecific competition (*κ*), and the vertical axis denotes the IRNI. The solid line and the dashed line represent the IRNI of the *K*-strategy and *r*-strategy, respectively. The curves are given by Eqs (F.8), (F.9) and (F.15) at Text F in S1 File. The IRNI of the *r*-strategy is always greater than that of the others based on its definition. The domains of both the specialists and generalists represent the *K*-strategy because the *r*-strategy is independent of intraspecific competition. The parameters used are the same as those used for Fig 2.

**Fig 4:**
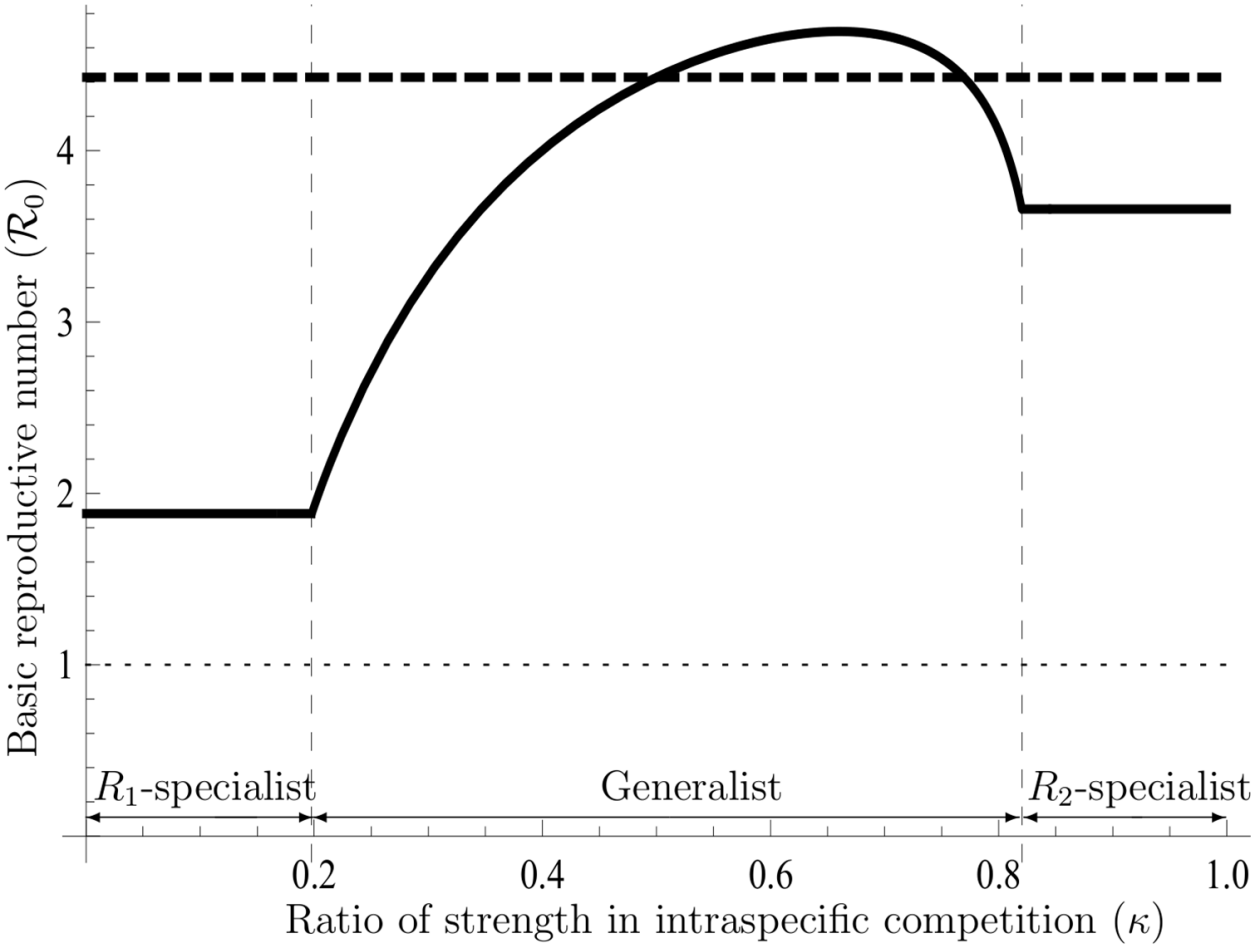
The potential basic reproductive numbers in the *r*- and *K*-strategies. This figure illustrates $R_{0}$ for both strategies in the absence of density effects. The horizontal axis shows the ratio of the strength in the intraspecific competition (*κ*), and the vertical axis represents $R_{0}$ under Γ_*t*_ = 0. The solid and the dashed lines represent the same entities as those in Fig 3, and the parameters used are also the same. It is shown that the *K*-strategy of $R_{0}$ is not always higher than the other. Therefore, the basic reproductive number of the *K*-strategy potentially depends on the ratio of the strength in the intraspecific competition.

**Fig 5:**
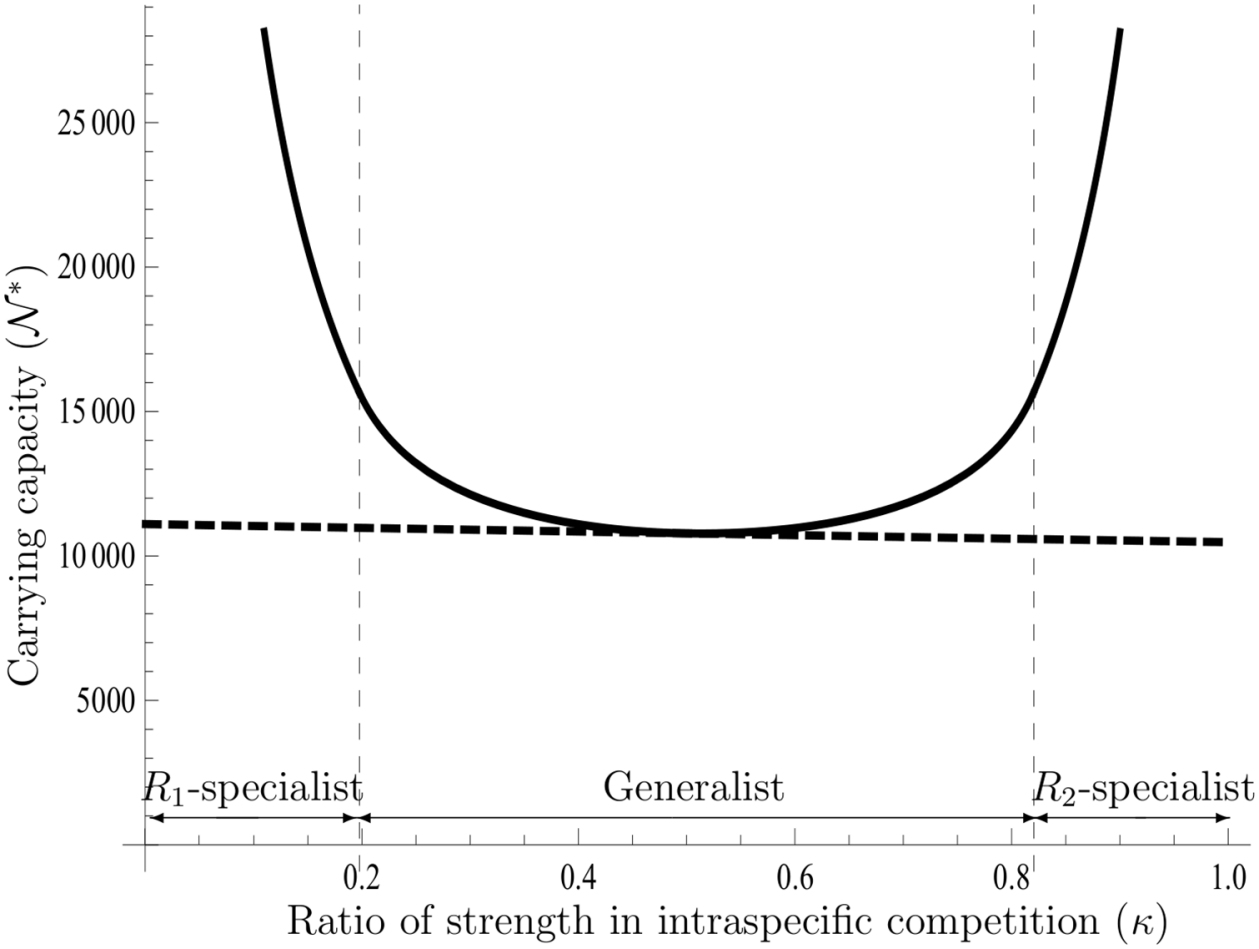
Sizes of the carrying capacity in both *r*/*K* strategies. The horizontal axis represents the ratio of the strength in the intraspecific competition (*κ*), and the vertical axis represents the size of the carrying capacity $N^{*}$. The solid and the dashed lines represent the same entities as those in Fig 3, and the parameters used are also the same. Each vertical dashed line represents the boundary between the specialists and the generalists. This figure shows that the *K*-strategy always has greater carrying capacity than the other strategies, as demonstrated by Eq (32).

Substituting Eqs (35), (37) and (51) into Eq (52), we obtain the optimal risk hedging ${\tilde{v}}_{S}$ under ρ* < 1, such that

$$\begin{array}{c} \tilde{v}\left(\rho^{*},\Gamma \right)=\max\left\{\min\left\{\frac{\sigma_{1}^{2}}{\sigma_{1}^{2}+\sigma_{2}^{2}}-\frac{b_{1}-b_{2}+\left(1-2\kappa \right)\Gamma }{\left(\sigma_{1}^{2}+\sigma_{2}^{2}\right)\left(1-\rho^{*}\right)},1\right\},0\right\}, \end{array}$$ (53)

(see Text F in S1 File). Eq (53) implies that the *K*-strategy ${\tilde{v}}_{K}(\rho^{*})$ is biased towards either one of the two strategies of utilization at the border *κ* = 0.5. Yet, in *κ* = 0.5, due to a counterbalance between competition rates in both resources, the *r* and *K* strategies have an identical utilization ratio $\tilde{v}$. In another situation (*κ* ≠ 0.5), the *K*-strategy can evolve all patterns of utilization ratios in accordance with the value of *κ*, which is different from the *r*-strategy (see Fig 2); for instance, the evolution of *R*_2_-specialists never occurs in *r*-selection [9]. Accordingly, it is also possible that the *K*-strategy potentially has smaller values of IRNI and $R_{0}$ than the other ${\tilde{v}}_{r}(\tilde{\rho })$ (see Figs 3 and 4). This result suggests that the use of IRNI and $R_{0}$ as the fitness must be restricted. As shown in Eq (32), the *K*-strategy always has a larger carrying capacity than any other strategy even if the IRNI and $R_{0}$ are potentially smaller than those obtained with other strategies (cf. Fig 5). Note that those simulations are collected to illustrate shapes of functions and properties (cf. Figs 1–5); these are exactly given: Eqs (32), (53), and Eq (F.15) at Text F in S1 File. For this reason, comparison with the exact values of those parameters in the nature is not so important to the purpose of analysis.

Because augmenting EES reduces the effect of internal stochasticity on the carrying capacity (see Eq (44)), Eq (53) has the following limit

$$\begin{array}{c} \lim_{\rho^{*}↑1}{\tilde{v}}_{K}\left(\rho^{*}\right)=\left\{\begin{array}{cc} 1 & \kappa >\kappa_{0}\\ 0 & \kappa <\kappa_{0} \end{array}\right.\;\left(\kappa_{0}:=\frac{1}{1+\frac{b_{2}-\mu_{0}}{b_{1}-\mu_{0}}}\right). \end{array}$$ (54)

If *κ* = *κ*_0_, an adaptive strategy does not exist because all strategies have identical carrying capacity. It is also shown that at the individual scale, Eq (31) becomes equal for all *v* ∈ [0, 1]. On the other hand, when *σ*_2_ = 0, we obtain an identical result from the deterministic case, such as $\lim_{\sigma_{1}↓0}\tilde{v}(\rho^{*},\Gamma )$. Generalists do not evolve in higher EES and in the absence of internal stochasticity. Therefore, risk hedging is important for the persistence of the species with smaller EES than those under internal stochasticity.

## Discussion

The central point of *r*/*K*-selection theory is *K*-selection. This study proposes an interpretation of the benefits of *K*-strategies by adapting concepts of ESS to structured population models. Natural selection operates essentially through an identical parameter Eq (27) in both *r*- and *K*-selection. This parameter represents the reproductive contribution of all cohorts to the population dynamics. Our specific example raises the possibility that the *K*-strategy leads to opposite traits than that obtained using the *r*-strategy as Pianka suggested [3]. However, it also indicates that internal stochasticity is able to induce strategies that are different from Pianka’s classification. The example is a *K*-strategy with a reproductive number identical to that of the *r*-strategy and with an IRNI lower than that of the other strategies, which is not included in Pianka’s classification (Cf. Figs 3 and 4). The benefit of the *K*-strategy for individuals is that the population is not invaded by mutants with any other strategies. On the other hand, our study suggests that several traits of the *K*-strategies in Pianka’s classification are not essentially adaptive strategies but are by-products of adaptation. Although recent studies do not focus on examining Pianka’s classifications, it is still significant in any ecological research that considers adaptive traits of life history in the presence of density effects [4]. Various life histories of organisms are thought to result from adaptation to intraspecific competition. Both *r*- and *K*-strategies arise from identical consequences, of which individuals maximize their reproductive contribution to the population dynamics (Eq (11)) under different magnitudes of density effects. Density effects are, however, unable to impair the *r*-strategy as a result of intraspecific competition. Consequently, species evolve the strategy that seems to be intuitively disadvantageous (e.g., altricity or low basic reproductive number). Though it seems that it is difficult for the strategy to evolve under *r*-selection, these disadvantages are not essential for the adaptive strategy in *K*-selection. Our results present a different viewpoint in *r*/*K* selection theory. As mentioned above, the reproductive traits of *K*-strategies are not always the purpose of adaptation, but they are by-products caused by at least the adaptive strategies. A significant criterion for the *K*-strategy is whether a strategy becomes ESS in terms of the carrying capacity or not; it should have the highest level of intraspecific competition $\tilde{\Gamma }$. Then, the individual lifespan, reproductive number, maturity, and other vital rates are determined by what the population density affects in their life history. Density effects, thus, are able to influence not only mortality but also the other components of life history, and they alter the statistical characteristics of the total contributions of all cohorts Eq (28). Various traits of life histories, besides precocity and prolificacy, might arise due to the complicated system of density effects. In addition, this study proposes how we analyze the evolution of life histories under multiple density effects with internal stochasticity.

By contrast, conventional *r*-selection theory has thought that the evolution of the *r*-strategy occurs with high natural mortality as long as the intraspecific interaction is negligible. The *r*-strategy ${\tilde{v}}_{r}$ maximizes the IRNI at a sufficiently small population density; nevertheless, Eq (31) indicates that the population is eventually dominated by the *K*-strategy ${\tilde{v}}_{K}$. Accordingly, the evolution of the *r*-strategy is unlikely to occur under assumptions (i-iii). For example, if an adaptive strategy is independent of the density effects, the *r*-strategy might remain as ESS at all population sizes because it combines both strategies (${\tilde{v}}_{r}={\tilde{v}}_{K}$), such as *κ* = 0.5 in Eq (49). In contrast, the *r*-strategy appears to confer an advantage over a significant disturbance because of its higher population growth rate. As discussed in previous studies on this subject, the *r*-strategy possibly serves as plasticity with respect to the reduction of the population size by external stochasticity. Eqs (25) and (26) can only provide local adaptive control at each density effect level Γ*, which does not mean prohibiting a species from evolving to have plastic control at each population size. Therefore, the *r*-strategy is thought to be incorporated into plastic control, which switches from the *r*-strategy to the *K*-strategy associated with an increase in population size (such as the phase variation in migratory locusts [1]).

### Conclusion

This study proposes unifying the theory on optimal life schedule and nonlinear structured population models using a function representing the reproductive contribution of each cohort. The function provides a solution to two equations (Eqs (25) and (26)). Applying these equations to *r*/*K*-selection theory, we can clarify the relationship between the individual scale and population scale in evolution. *r*/*K*-selection theory consists of a specific environment, where the population growth converges to a unique carrying capacity (Cf. assumptions (i)-(iii)). In nature, all habitats are not necessarily identical to such kinds of environment. Theoretical studies based on dynamical systems predict that density effects can generate multiple equilibria and periodic, or chaotic behaviors of population growth [15]. Furthermore, population dynamics are also thought to be affected by external stochasticity [16]. Because Eqs (25) and (26) can address the adaptive strategy surrounding each stable equilibrium, we can apply them to models that contain multiple equilibria. In this case, each stable equilibrium is supposed to give rise to the adaptive strategy that can be regarded as speciation such as Adaptive Dynamics theory. The difference between our method and AD is that it treats not the trait value but the dynamical controls of life history as the object in natural selection. This means that our method releases the specific adaptive phenotype from the concept of trait value in terms of lumping various life strategies in quantitative genetics. Moreover, we can derive a novel fitness function from three ingredients of life history consisting of state transition rate *dX*_*a*_, fertility *F*(⋅), and mortality *μ*(⋅) via Eq (25). Depending on density effect of each state (e.g., *γ*^*m*^(⋅)), the population growth do not always have stable equilibria generally as which theoreticians predicted. In this case, we have few methods to handle it. The similar matter is also argued in the presence of external stochasticity [16–18]. Many species are thought to have various population dynamics besides the logistic type and to also be affected by external stochasticity. A further extension of this framework will be required that can account for adaptive strategies under unstable or fluctuating population dynamics in future work. Consequently, we hope that this study will become a basis for systematization in evolutionary theory addressing the effects described above.

## Supporting Information

S1 File Text A, Derivation of the Non-linear Partial Differential Equation. Text B, General stability analysis. Text C, Derivation of the HJB equation and stationary control. Text D, Analysis of the resource acquisition competition model. Text E, The mature age density of the resource acquisition competition model. Text F, Analysis of the optimal utilization.
(PDF)

## References

[1] Uvarov BP. A revision of the genus Locusta, L.(= Pachytylus, Fieb.), with a new theory as to the periodicity and migrations of locusts. Bulletin of Entomological Research. 1921;12(02):135–163. 10.1017/S0007485300044989

[2] MacArthur RH, Wilson EO. The theory of island biogeography. Princeton Univ Pr; 1967.

[3] Pianka ER. On r-and K-selection. American naturalist. 1970;p. 592–597. 10.1086/282697

[4] Reznick D, Bryant MJ, Bashey F. r-and K-selection revisited: the role of population regulation in life-history evolution. Ecology. 2002;83(6):1509–1520. 10.1890/0012-9658(2002)083[1509:RAKSRT]2.0.CO;2

[5] Engen S, Lande R, Sæther BE. A Quantitative Genetic Model of r-and K-Selection in a Fluctuating Population. The American Naturalist. 2013;181(6):725–736. 10.1086/670257 23669536

[6] Leon JA. Life histories as adaptive strategies. Journal of theoretical Biology. 1976;60(2):301–335. 10.1016/0022-5193(76)90062-X 957718

[7] Mylius SD, Diekmann O. On evolutionarily stable life histories, optimization and the need to be specific about density dependence. Oikos. 1995;p. 218–224. 10.2307/3545651

[8] Oizumi R, Takada T. Optimal life schedule with stochastic growth in age-size structured models: theory and an application. Journal of Theoretical Biology. 2013;323:76–89. 10.1016/j.jtbi.2013.01.020 23391431

[9] Oizumi R. Unification Theory of Optimal Life Histories and Linear Demographic Models in Internal Stochasticity. PLOS ONE. 2014;9(6):e98746 10.1371/journal.pone.0098746 24945258 PMC4063715

[10] Langlais M. Large time behavior in a nonlinear age-dependent population dynamics problem with spatial diffusion. Journal of mathematical biology. 1988;26(3):319–346. 10.1007/BF00277394 3411256

[11] Webb G. Population models structured by age, size, and spatial position In: Structured Population Models in Biology and Epidemiology. Springer; 2008 p. 1–49.

[12] Lv Q, Pitchford JW. Stochastic von Bertalanffy models, with applications to fish recruitment. Journal of theoretical biology. 2007;244(4):640–655. 10.1016/j.jtbi.2006.09.009 17055532

[13] Gurtin ME, Maccamy RC. Non-linear age-dependent population dynamics. Archive for Rational Mechanics and Analysis. 1974;54(3):281–300. 10.1007/BF00250793

[14] Yong J, Zhou XY. Stochastic controls: Hamiltonian systems and HJB equations. vol. 43 Springer Verlag; 1999.

[15] Magal P, Ruan S. Center manifolds for semilinear equations with non-dense domain and applications to Hopf bifurcation in age structured models. vol. 202 American Mathematical Society; 2009.

[16] Salguero-Gómez R, De Kroon H. Matrix projection models meet variation in the real world. Journal of Ecology. 2010;98(2):250–254. 10.1111/j.1365-2745.2009.01635.x

[17] Lauenroth WK, Adler PB. Demography of perennial grassland plants: survival, life expectancy and life span. Journal of Ecology. 2008;96(5):1023–1032. 10.1111/j.1365-2745.2008.01415.x

[18] Morris WF, Pfister CA, Tuljapurkar S, Haridas CV, Boggs CL, Boyce MS, et al Longevity can buffer plant and animal populations against changing climatic variability. Ecology. 2008;89(1):19–25. 10.1890/07-0774.1 18376542

