# Supplementary material for: Reconsideration of r/K Selection Theory Using Stochastic Control Theory and Nonlinear Structured Population Models
Source: PLoS One. 2016 Jun 23;11(6):e0157715. doi: 10.1371/journal.pone.0157715 (PMC4919082; doi:10.1371/journal.pone.0157715)
Supplement: S1 File — Text A, Derivation of the Non-linear Partial Differential Equation. Text B, General stability analysis. Text C, Derivation of the HJB equation and stationary control. Text D, Analysis of the resource acquisition competition model. Text E, The mature age density of the resource acquisition competition model. Text F, Analysis of the optimal utilization. (PDF) [file pone.0157715.s001.pdf]

Supporting Information

Text A

Derivation of the Non-linear Partial Differential Equation

Suppose that $f(y)$ is a second-order differentiable function and becomes zero at the boundary of A (i.e., $f(y) \in C_0^2(A)$). From Eq. (5), we consider

$$\int_A dy\ f(y)\, P_{t+\varepsilon}(a+\varepsilon, x \to y) = \int_A dy\ f(y) \int_A d\xi\ K_{\varepsilon,t}(\xi \to y)\, P_t(a, x \to \xi). \tag{A.1}$$

Then, fixing t and a, all the functions above with respect to t and a are regarded as functions of ε such that

$$\bar{P}_\varepsilon(x \to y) = P_{t+\varepsilon}(a+\varepsilon, x \to y) \tag{A.2}$$
$$\bar{K}_\varepsilon(x \to y) = K_{\varepsilon,t}(x \to y), \tag{A.3}$$

When

$$u_\varepsilon(x) := \int_A dy\ f(y)\, \bar{K}_\varepsilon(x \to y),$$

the RHS of Eq. (A.1) has a changed order of integral in terms of ξ and y as follows:

$$\int_A dy\ f(y)\, \bar{P}_\varepsilon(x \to y) = \int_A d\xi\ u_\varepsilon(\xi)\, \bar{P}_0(x \to \xi). \tag{A.4}$$

The derivative of both sides with respect to ε is

$$\int_A dy\ f(y)\, \frac{\partial}{\partial\varepsilon}\bar{P}_\varepsilon(x \to y) = \int_A d\xi\ \frac{\partial}{\partial\varepsilon}u_\varepsilon(\xi)\, \bar{P}_0(x \to \xi). \tag{A.5}$$

From the definition of the projection function $\bar{K}_\varepsilon(x \to y)$, $u_\varepsilon(\xi)$ is interpreted as the expectation generated by the statistics of Eqs. (1) and (3) such that

$$u_\varepsilon(\xi) = \mathbb{E}_\xi\left[f\left(\bar{X}_\varepsilon\right)\exp\left\{-\int_0^\varepsilon ds\ \mu\left(\bar{X}_\varepsilon, v, \Gamma_{t+s}\right)\right\}\right], \tag{A.6}$$

where

$$\bar{X}_\varepsilon = X_\varepsilon(t+\varepsilon).$$

This expression satisfies

$$\begin{cases} \dfrac{\partial}{\partial\varepsilon}u_\varepsilon(\xi) = -\bar{\mathcal{H}}_\xi^v(\Gamma_{t,x})\, u_\varepsilon(\xi) \\ \bar{\mathcal{H}}_\xi^v(\Gamma_{t,x}) := -\displaystyle\sum_{j=1}^d g_j(\xi, v, \Gamma_{t,x})\frac{\partial}{\partial\xi^j} - \frac{1}{2}\sum_{j,j'=1}^d c_{jj'}(\xi, v, \Gamma_{t,x})\frac{\partial^2}{\partial\xi^j\partial\xi^{j'}} + \mu(\xi, v, \Gamma_{t,x}) \\ u_0(\xi) = f(\xi), \end{cases} \tag{A.7}$$

which is called the Feynman-Kac formula [1]. This rewrites the RHS of Eq. (A.5) as follows:

$$\int_A d\xi\ \frac{\partial}{\partial\varepsilon}u_\varepsilon(\xi)\, \bar{P}_0(x \to \xi) = -\int_A d\xi\ \bar{\mathcal{H}}_\xi^v(\Gamma_{t,x})\, u_\varepsilon(\xi)\, \bar{P}_0(x \to \xi) \tag{A.8}$$

By iterative integration by parts in the RHS of the equation above, we obtain

$$\int_A d\xi\ \frac{\partial}{\partial\varepsilon}u_\varepsilon(\xi)\, \bar{P}_0(x \to \xi) = -\int_A d\xi\ u_\varepsilon(\xi)\, \mathcal{H}_\xi^v(\Gamma_{t,x})\, \bar{P}_0(x \to \xi), \tag{A.9}$$

from the property of $f(y)$. Taking the limit of $\varepsilon \downarrow 0$ in Eq. (A.5), we get

$$\int_A dy\; f(y)\left[\frac{\partial}{\partial t}+\frac{\partial}{\partial a}\right]\bar{P}_0(x\to y) = -\int_A dy\; f(y)\,\mathcal{H}_\xi^v(\Gamma_{t,x})\,\bar{P}_0(x\to\xi). \tag{A.10}$$

Here, we use

$$\frac{\partial}{\partial\varepsilon}\bar{P}_\varepsilon(x\to y) = \left[\frac{\partial}{\partial t}+\frac{\partial}{\partial a}\right]P_{t+\varepsilon}(a+\varepsilon, x\to y),$$

and

$$\lim_{\varepsilon\downarrow 0}\bar{K}_\varepsilon(\xi\to y) = \delta^d(\xi - y).$$

Therefore, Eq. (A.10) is held for all $f(y)$ as long as the population density satisfies Eq. (8).

Text B

General stability analysis

Let the perturbation variables be

$$\varphi_t(a, x\to y) := P_t(a, x\to y) - P^*(a, x\to y), \qquad \vartheta_t^m := \Gamma_t^m - \Gamma_x^{m*}.$$

Then, the first-order derivative of the perturbation follows

$$\begin{cases}\left[\frac{\partial}{\partial t}+\frac{\partial}{\partial a}\right]\varphi_t(a,x\to y) = -\mathcal{H}_y^{0,v}\varphi_t(a,x\to y) - \sum_{m=1}^{M}\vartheta_t^m\mathcal{H}_{m,y}^{1,v}P^*(a,x\to y)\\ \mathcal{H}_y^{0,v} := \sum_{j=1}^{d}\frac{\partial}{\partial y^j}g_j(y,v,\Gamma_x^*) - \frac{1}{2}\sum_{j,j'=1}^{d}\frac{\partial^2}{\partial y^j\partial y^{j'}}c_{jj'}(y,v,\Gamma_x^*) + \mu(y,v,\Gamma_x^*)\\ c_{jj'}(y,v,\Gamma_x^*) := \sum_{k=1}^{N}\sigma_{jk}(y,v,\Gamma_x^*)\,\sigma_{j'k}(y,v,\Gamma_x^*)\\ \mathcal{H}_{m,y}^{1,v} := \frac{\partial}{\partial\Gamma^m}\left(\mathcal{H}_y^{0,v}\right)\Big|_{\Gamma=\Gamma_x^*}\\ \varphi_t(0,x\to y) = n_t^\dagger(x)\,\delta^d(x-y)\\ n_t^\dagger(x) := n_t(x) - n^*(x) = \int_0^\alpha da\int_A dy\left[F(y,\Gamma_x^*) + \sum_{m=1}^{M}q_m\gamma^m(a,y)\right]\varphi_t(a,x\to y)\\ \varphi_0(a,x\to y) = \mathcal{P}(a,x,y) - P^*(a,x\to y)\\ q_m = \int_0^\alpha da\int_A dy\frac{\partial}{\partial\Gamma^m}F(y,\Gamma)\Big|_{\Gamma=\Gamma_x^*}P^*(a,x\to y).\end{cases} \tag{B.1}$$

Using a characteristic curve of the above equation, we get

$$\frac{\partial}{\partial\varepsilon}\bar{\varphi}_\varepsilon(x\to y) = -\mathcal{H}_y^{0,v}\bar{\varphi}_\varepsilon(x\to y) - \sum_{m=1}^{M}\vartheta_\varepsilon^m\mathcal{H}_{m,y}^{1,v}P^*(\varepsilon,x\to y), \tag{B.2}$$

where

$$\bar{\varphi}_\varepsilon(x\to y) := \varphi_{t+\varepsilon}(a+\varepsilon, x\to y). \tag{B.3}$$

This equation has the following formal solution

$$\begin{aligned}\bar{\varphi}_\varepsilon (x \to y) &= \bar{n}_0^\dagger (x) \exp\left\{-\varepsilon \mathcal{H}_y^{0,v}\right\} \delta^d (x-y) \\ &- \sum_{m=1}^{M} \int_0^\varepsilon ds\ \vartheta_s^m \exp\left\{-(\varepsilon - s)\mathcal{H}_y^{0,v}\right\} \mathcal{H}_{m,y}^{1,v} P^* (s, x \to y) \qquad \text{(B.4)} \\ &= \bar{n}_0^\dagger (x) K_\varepsilon^{*,v} (x \to y) \\ &- \sum_{m=1}^{M} \int_0^\varepsilon ds\ \vartheta_s^m \int_A d\xi\ K_{\varepsilon - s}^{*,v} (\xi \to y) \mathcal{H}_{m,\xi}^{1,v} P^* (s, x \to \xi), \qquad \text{(B.5)}\end{aligned}$$

or

$$\begin{aligned}\bar{\varphi}_\varepsilon (x \to y) &= \exp\left\{-\varepsilon \mathcal{H}_y^{0,v}\right\} \varphi_0 (0, x \to y) \\ &- \sum_{m=1}^{M} \int_0^\varepsilon ds\ \vartheta_s^m \exp\left\{-(\varepsilon - s)\mathcal{H}_y^{0,v}\right\} \mathcal{H}_{m,y}^{1,v} P^* (s, x \to y) \qquad \text{(B.6)} \\ &= \int_A d\xi\ K_\varepsilon^{*,v} (\xi \to y) \varphi_0 (0, \xi) \\ &- \sum_{m=1}^{M} \int_0^\varepsilon ds\ \vartheta_s^m \int_A d\xi\ K_{\varepsilon - s}^{*,v} (\xi \to y) \mathcal{H}_{m,\xi}^{1,v} P^* (s, x \to \xi). \qquad \text{(B.7)}\end{aligned}$$

Therefore, setting a new coordinate

$$(t, a) \to \begin{cases} (t-a, 0) & t-a > 0, \\ (0, a-t) & a-t > 0, \end{cases} \tag{B.8}$$

the solution of Eq. (B.1) is

$$\varphi_t (a, x \to y) = \begin{cases} n_{t-a}^\dagger (x) K_a^{*,v} (x \to y) - \\ \sum_{m=1}^{M} \int_0^a ds\ \vartheta_{t-a+s}^m \int_A d\xi\ K_{a-s}^{*,v} (\xi \to y) \mathcal{H}_{m,\xi}^{1,v} P^* (s, x \to \xi) & t-a > 0 \\ \int_A d\xi\ K_t^{*,v} (\xi \to y) \varphi_0 (a-t, \xi) - \\ \sum_{m=1}^{M} \int_0^t ds\ \vartheta_s^m \int_A d\xi\ K_{t-s}^{*,v} (\xi \to y) \mathcal{H}_{m,\xi}^{1,v} P^* (a-t+s, x \to \xi) & a-t > 0. \end{cases} \tag{B.9}$$

Then, the density effect $\bar{\Gamma}_t^m$ becomes

$$\vartheta_t^m = \begin{cases} \int_0^t da\ n_{t-a}^\dagger (x) \nu_a^m (x) - \\ \sum_{m'=1}^{M} \int_0^t da \int_0^a ds\ \vartheta_{t-a+s}^{m'} \int_A d\xi\ \nu_{a-s}^m (\xi) \mathcal{H}_{m',\xi}^{1,v} P^* (s, x \to \xi) & t-a > 0 \\ \int_t^\alpha da \int_A d\xi\ \nu_t^m (\xi) \varphi_0 (a-t, x \to \xi) - \\ \sum_{m'=1}^{M} \int_t^\alpha da \int_0^t ds\ \vartheta_s^{m'} \int_A d\xi\ \nu_{t-s}^m (\xi) \mathcal{H}_{m',\xi}^{1,v} P^* (a-t+s, x \to \xi) & a-t > 0, \end{cases} \tag{B.10}$$

where

$$\nu_a^m (x) := \mathbb{E}_x^* \left[\gamma^m (a, X_a) S (a, \Gamma_x^*)\right] \tag{B.11}$$

and

$$S (a, \Gamma_x^*) := \exp\left\{-\int_0^a d\tau \mu (X_\tau, v, \Gamma_x^*)\right\}.$$

Substituting Eq. (B.9) into the boundary condition of Eq. (B.1) , we obtain the Volterra integral equation

$$\begin{aligned}n_t^\dagger(x) = G_t(x) + \int_0^t da\ n_{t-a}^\dagger(x)\, u_a^*(x) \\ - \sum_{m=1}^{M}\int_A d\xi \int_t^\alpha da \int_0^t ds\ \vartheta_s^m u_{t-s}^*(\xi)\, \mathcal{H}_{m,\xi}^{1,v} P^*(a-t+s, x \to \xi) \\ - \sum_{m=1}^{M}\int_A d\xi \int_0^t da \int_0^a ds\ \vartheta_{t-a+s}^m u_{a-s}^*(\xi)\, \mathcal{H}_{m,\xi}^{1,v} P^*(s, x\to\xi) + \sum_{m=1}^{M} q_m \vartheta_t^m\end{aligned} \tag{B.12}$$

$$\begin{aligned}G_t(x) :&= \int_t^\alpha da \int_A d\xi \varphi_0(a-t, x, \xi)\, u_t^*(\xi) \\ u_a^*(x) :&= \mathbb{E}_x^*\left[F(X_a, \Gamma_x^*)\, S(a, \Gamma_x^*)\right] = \int_A dy\ F(y, \Gamma_x^*)\, K_a^{*,v}(x \to y)\end{aligned} \tag{B.13}$$

Additionally, changing the variable of Eq. (B.12) as follows,

$$\begin{cases} a-s \\ t-s \end{cases} \to \tau, \tag{B.14}$$

we have

$$\begin{aligned}n_t^\dagger(x) &= G_t(x) + \int_0^t da\ n_{t-a}^\dagger(x)\, u_a^*(x) \\ &- \sum_{m=1}^{M}\int_A d\xi \int_t^\alpha da \int_0^t d\tau\ \vartheta_{t-\tau}^m u_\tau^*(\xi)\, \mathcal{H}_{m,\xi}^{1,v} P^*(a-\tau, x\to\xi) \\ &- \sum_{m=1}^{M}\int_A d\xi \int_0^t da \int_0^a d\tau\ \vartheta_{t-\tau}^m u_\tau^*(\xi)\, \mathcal{H}_{m,\xi}^{1,v} P^*(a-\tau, x\to\xi) + \sum_{m=1}^{M} q_m\vartheta_t^m \\ &= G_t(x) + \int_0^t da\ n_{t-a}^\dagger(x)\, u_a^*(x) \\ &- \sum_{m=1}^{M}\int_A d\xi \int_0^t d\tau \int_t^\alpha da\ \vartheta_{t-\tau}^m u_\tau^*(\xi)\, \mathcal{H}_{m,\xi}^{1,v} P^*(a-\tau, x\to\xi) \\ &- \sum_{m=1}^{M}\int_A d\xi \int_0^t d\tau \int_\tau^t da\ \vartheta_{t-\tau}^m u_\tau^*(\xi)\, \mathcal{H}_{m,\xi}^{1,v} P^*(a-\tau, x\to\xi) + \sum_{m=1}^{M} q_m\vartheta_t^m,\end{aligned} \tag{B.15}$$

i.e.,

$$\begin{aligned}n_t^\dagger(x) =&G_t(x) + \int_0^t da\ n_{t-a}^\dagger(x)\, u_a^*(x) \\ &- \sum_{m=1}^{M}\int_A d\xi \int_0^t d\tau \int_\tau^\alpha da\ \vartheta_{t-\tau}^m u_\tau^*(\xi)\, \mathcal{H}_{m,\xi}^{1,v} P^*(a-\tau, x\to\xi) + \sum_{m=1}^{M} q_m\vartheta_t^m.\end{aligned} \tag{B.16}$$

On the other hand, we can rewrite Eq. (B.10) of ϑ_t^m as follows.

$$\begin{aligned}\vartheta_t^m =& H_t^m(x) + \int_0^t da\ n_{t-a}^{\dagger}(x)\,\nu_a^m(x) \\ &- \sum_{m'=1}^{M}\int_A d\xi \int_0^t d\tau \int_\tau^\alpha da\ \vartheta_{t-\tau}^{m'} \nu_\tau^m(\xi)\,\mathcal{H}_{m',\xi}^{1,v} P^*(a-\tau, x\to\xi),\end{aligned} \tag{B.17}$$

where

$$H_t^m(x) = \int_t^\alpha da \int_A d\xi\ \nu_t^m(\xi)\,\varphi_0(a-t,\xi).$$

Thus, setting

$$U_t(x) := \begin{pmatrix} n_t(x) \\ \theta_t^1 \\ \vdots \\ \theta_t^M \end{pmatrix},\ B = \begin{pmatrix} 0 & q_1 & \cdots & q_M \\ 0 & 0 & \cdots & 0 \\ \vdots & \vdots & \ddots & \vdots \\ 0 & \cdots & & 0 \end{pmatrix},\ \mathcal{F}_t(x) := \begin{pmatrix} G_t(x) \\ H_t^1(x) \\ \vdots \\ H_t^M(x) \end{pmatrix},$$

$$A_\tau(x) :=$$

$$\begin{pmatrix} u_\tau^*(x) & -\int_\tau^\alpha da\int_A d\xi\ u_\tau^*(\xi)\mathcal{H}_{1,\xi}^{1,v}P^*(a-\tau,x\to\xi) & \cdots & -\int_\tau^\alpha da\int_A d\xi\ u_\tau^*(\xi)\mathcal{H}_{M,\xi}^{1,v}P^*(a-\tau,x\to\xi) \\ \nu_\tau^1(x) & -\int_\tau^\alpha da\int_A d\xi\ \nu_\tau^1(\xi)\mathcal{H}_{1,\xi}^{1,v}P^*(a-\tau,x\to\xi) & \cdots & -\int_\tau^\alpha da\int_A d\xi\ \nu_\tau^1(\xi)\mathcal{H}_{M,\xi}^{1,v}P^*(a-\tau,x\to\xi) \\ \vdots & \vdots & \ddots & \vdots \\ \nu_\tau^M(x) & -\int_\tau^\alpha da\int_A d\xi\ \nu_\tau^M(\xi)\mathcal{H}_{1,\xi}^{1,v}P^*(a-\tau,x\to\xi) & \cdots & -\int_\tau^\alpha da\int_A d\xi\ \nu_\tau^M(\xi)\mathcal{H}_{M,\xi}^{1,v}P^*(a-\tau,x\to\xi) \end{pmatrix},$$

we have from Eqs.(B.16) and (B.17) the following Volterra integral equation.

$$U_t(x) = \mathcal{F}_t(x) + \int_0^t A_\tau(x) U_{t-\tau}(x) d\sigma + BU_t(x)$$

Rearranging it gives

$$U_t(x) = (\mathbf{I}-B)^{-1}\mathcal{F}_t(x) + \int_0^t (\mathbf{I}-B)^{-1} A_\tau(x) U_{t-\tau}(x) d\sigma,$$

where $\mathbf{I}$ denotes the identity matrix. Then, it follows from the Paley-Wiener theorem (see [2]) that the equilibrium is asymptotically stable if and only if all roots λ of

$$\det\left(\mathbf{I} - B - \hat{A}_\lambda(x)\right) = 0 \tag{B.18}$$

have only negative real parts. Here,

$$\hat{A}_\lambda(x) := \int_0^\infty d\tau \exp\{-\lambda\tau\} A_\tau(x)$$

denotes the Laplace transform of $A_\tau(x)$. Because

$$\int_0^\infty d\tau\ \exp\{-\lambda\tau\}\int_\tau^\infty da\ g(\tau)\,h(a-\tau) = \int_0^\infty du \int_0^\infty d\tau\ \exp\{-\lambda\tau\}\,g(\tau)\,h(u),$$

we have

$$\hat{A}_\lambda(x) := \begin{pmatrix} \psi_\lambda^*(x) & -\int_A d\xi \psi_\lambda^*(\xi)\mathcal{H}_{1,\xi}^{1,v}\hat{P}^*(x\to\xi) & \cdots & -\int_A d\xi \psi_\lambda^*(\xi)\mathcal{H}_{M,\xi}^{1,v}\hat{P}^*(x\to\xi) \\ I_\lambda^1(x) & -\mathscr{D}_{\lambda,1,1} & \cdots & -\mathscr{D}_{\lambda,M,1} \\ \vdots & \vdots & \ddots & \vdots \\ I_\lambda^M(x) & -\mathscr{D}_{\lambda,1,M} & \cdots & -\mathscr{D}_{\lambda,M,M} \end{pmatrix},$$

where

$$\psi_\lambda^*(x) := \int_0^\alpha da\ \exp\{-\lambda a\}\, \mathbb{E}_x^*\left[F(X_a, \Gamma_x^*)\, S(a, \Gamma_x^*)\right] \tag{B.19}$$

$$I_\lambda^m(x) := \int_0^\alpha da \exp\{-\lambda a\}\, \mathbb{E}_x^*\left[\gamma^m(a, X_a)\, S(a, \Gamma_x^*)\right], \quad 1 \le m \le M \tag{B.20}$$

$$\hat{P}^*(x \to \xi) := \int_0^\alpha da\ P^*(a, x \to \xi) \tag{B.21}$$

$$\mathscr{D}_{\lambda,m',m} := \int_A d\xi\ I_\lambda^m(\xi)\, \mathcal{H}_{m',\xi}^{1,v} \hat{P}^*(x \to \xi), \quad 1 \le m, m' \le M. \tag{B.22}$$

Hence, setting

$$\begin{aligned}
\mathscr{Q}_m &:= \int_A d\xi \psi_\lambda^*(\xi)\, \mathcal{H}_{m,\xi}^{1,v} \hat{P}^*(x \to \xi) - q_m, \quad 1 \le m \le M \\
\mathscr{Q}_\lambda &:= (\mathscr{Q}_1, \cdots, \mathscr{Q}_m, \cdots, \mathscr{Q}_M) \\
\mathbf{D}_\lambda &:= (\mathscr{D}_{\lambda,m',m})_{1 \le m, m' \le M} \\
\mathcal{I}_\lambda &:= {}^t\left(I_\lambda^1(x), \cdots, I_\lambda^m(x), \cdots, I_\lambda^M(x)\right),
\end{aligned}$$

we have

$$\begin{aligned}
&\det\left(\mathbf{I} - B - \hat{A}_\lambda(x)\right) \\
&= \det\begin{pmatrix} 1 - \psi_\lambda^*(x) & \mathscr{Q}_1 & \cdots & \mathscr{Q}_M \\ -I_\lambda^1(x) & 1 + \mathscr{D}_{\lambda,1,1} & \cdots & \mathscr{D}_{\lambda,M,1} \\ \vdots & \vdots & \ddots & \vdots \\ -I_\lambda^M(x) & \mathscr{D}_{\lambda,1,M} & \cdots & 1 + \mathscr{D}_{\lambda,M,M} \end{pmatrix} \\
&= \det\begin{pmatrix} 1 - \psi_\lambda^*(x) & \mathscr{Q}_\lambda \\ -\mathcal{I}_\lambda & \mathbf{I} + \mathbf{D}_\lambda \end{pmatrix} \\
&= (1 - \psi_\lambda^*(x)) \det(\mathbf{I} + \mathbf{D}_\lambda) + I_\lambda^1(x) \det\begin{pmatrix} \mathscr{Q}_\lambda \\ (\mathbf{I} + \mathbf{D}_\lambda)_2 \\ \vdots \\ (\mathbf{I} + \mathbf{D}_\lambda)_M \end{pmatrix} \\
&+ I_\lambda^2(x) \det\begin{pmatrix} (\mathbf{I} + \mathbf{D}_\lambda)_1 \\ \mathscr{Q}_\lambda \\ \vdots \\ (\mathbf{I} + \mathbf{D}_\lambda)_M \end{pmatrix} + \cdots + I_\lambda^M(x) \det\begin{pmatrix} (\mathbf{I} + \mathbf{D}_\lambda)_1 \\ (\mathbf{I} + \mathbf{D}_\lambda)_2 \\ \vdots \\ \mathscr{Q}_\lambda \end{pmatrix},
\end{aligned} \tag{B.23}$$

where $(\mathbf{I} + \mathbf{D}_\lambda)_i$ denotes the i-th component when we write the matrix as a column vector. Recalling that

$$\left(\mathscr{Q}_\lambda (\mathbf{I} + \mathbf{D}_\lambda)^{-1}\right)_i = \frac{1}{\det(\mathbf{I} + \mathbf{D}_\lambda)} \det\begin{pmatrix} (\mathbf{I} + \mathbf{D}_\lambda)_1 \\ \vdots \\ (\mathbf{I} + \mathbf{D}_\lambda)_{i-1} \\ \mathscr{Q}_\lambda \\ (\mathbf{I} + \mathbf{D}_\lambda)_{i+1} \\ \vdots \\ (\mathbf{I} + \mathbf{D}_\lambda)_M \end{pmatrix}, \quad 1 \le i \le M,$$

we have from Eq. (B.23) that

$$\det\left(\mathbf{I} - B - \hat{A}_\lambda(x)\right) = \left\{1 - \psi_\lambda^*(x) + \mathscr{Q}_\lambda (\mathbf{I} + \mathbf{D}_\lambda)^{-1} \mathcal{I}_\lambda\right\} \det(\mathbf{I} + \mathbf{D}_\lambda).$$

Hence, condition Eq. (B.18) leads to the following characteristic equation:

$$1 = \psi^*_\lambda(x) - \mathscr{Q}_\lambda(\mathbf{I} + \mathbf{D}_\lambda)^{-1}\mathcal{I}_\lambda =: \Psi_\lambda. \tag{B.24}$$

Text C

Derivation of the HJB equation

Let $\tilde{w}_{\lambda,a}(x,\Gamma)$ be the following function described by the age represented in descending order ($a \to \alpha - a$);

$$\begin{aligned} &\tilde{w}_{\lambda,a}(x,\Gamma) \\ &:= \sup_{v\in\mathcal{V}}\left\{\exp\{-\lambda(\alpha-a)\}\mathbb{E}_x\left[F(X_{\alpha-a},\Gamma)\exp\left\{-\int_0^{\alpha-a}d\tau\ \mu(X_\tau,v_\tau,\Gamma)\right\}\right]\right\}. \end{aligned} \tag{C.1}$$

Then, this function (referred to as the value function) follows Bellman's principle:

$$\begin{aligned} &\tilde{w}_{\lambda,a_0}(x,\Gamma) \\ &= \sup_{v\in\mathcal{V}}\left\{\exp\{-\lambda(a-a_0)\}\mathbb{E}_x\left[\tilde{w}_{\lambda,a}(X_{a-a_0},\Gamma)\exp\left\{-\int_0^{a-a_0}d\tau\ \mu(X_\tau,v_\tau,\Gamma)\right\}\right]\right\}, \end{aligned} \tag{C.2}$$

where $0 \le a_0 \le a \le \alpha$.This equation implies that the value function from a_0 to a corresponds to the value function $\tilde{w}_{\lambda,a_0}(x,\Gamma)$ from a_0 to α. Setting

$$\mathcal{E}_{\lambda,a}(\Gamma) := \exp\{-\lambda a\}\exp\left\{-\int_0^a d\tau\ \mu(X_\tau,v_\tau,\Gamma)\right\},$$

and using Ito's formula:

$$\begin{aligned} &d\left(\tilde{w}_{\lambda,a}(X_a,\Gamma)\mathcal{E}_{\lambda,a}(\Gamma)\right) = d\tilde{w}_{\lambda,a}(X_a,\Gamma)\mathcal{E}_{\lambda,a}(\Gamma) + \tilde{w}_{\lambda,a}(X_a,\Gamma)d\mathcal{E}_{\lambda,a}(\Gamma) \\ &= \frac{\partial}{\partial a}\tilde{w}_{\lambda,a}(X_a,\Gamma)\mathcal{E}_{\lambda,a}(\Gamma)da \\ &+ \left(\sum_{j=1}^d g_j(X_a,v,\Gamma)\frac{\partial}{\partial x^j} + \frac{1}{2}\sum_{j=1}^d\sum_{j'=1}^d c_{j,j'}(X_a,v,\Gamma)\frac{\partial^2}{\partial x^j\partial x^{j'}}\right)\tilde{w}_{\lambda,a}(X_a,\Gamma)\mathcal{E}_{\lambda,a}(\Gamma)da \\ &- (\mu(X_a,v,\Gamma)+\lambda)\tilde{w}_{\lambda,a}(X_a,\Gamma)\mathcal{E}_{\lambda,a}(\Gamma)da \\ &+ \sum_{k=1}^N \sigma_{jk}(X_a,v,\Gamma)\frac{\partial}{\partial x^j}\tilde{w}_{\lambda,a}(X_a,\Gamma)\mathcal{E}_{\lambda,a}(\Gamma)dB_a^k \\ &= \left(\frac{\partial}{\partial a} - \bar{\mathcal{H}}_x^v(\Gamma) - \lambda\right)\tilde{w}_{\lambda,a}(X_a,\Gamma)\mathcal{E}_{\lambda,a}(\Gamma)da \\ &\qquad + \sum_{k=1}^N \sigma_{jk}(X_a,v,\Gamma)\frac{\partial}{\partial x^j}\tilde{w}_{\lambda,a}(X_a,\Gamma)\mathcal{E}_{\lambda,a}(\Gamma)dB_a^k, \end{aligned}$$

Bellman's principle can be rewritten such that

$$\begin{aligned} 0 &= \sup_{v\in\mathcal{V}}\left\{\mathbb{E}_x\left[\tilde{w}_{\lambda,a}(X_{a-a_0},\Gamma)\mathcal{E}_{\lambda,a-a_0}(\Gamma)\right]\right\} - \tilde{w}_{\lambda,a_0}(x,\Gamma) \\ &= \sup_{v\in\mathcal{V}}\left\{\int_0^{a-a_0}d\tau\ \mathbb{E}_x\left[\left(\frac{\partial}{\partial a} - \bar{\mathcal{H}}_x^v(\Gamma) - \lambda\right)\tilde{w}_{\lambda,a_0+\tau}(X_\tau,\Gamma)\mathcal{E}_{\lambda,\tau}(\Gamma)\right]\right\}. \end{aligned} \tag{C.3}$$

Then, we use

$$\sum_{k=1}^{N}\mathbb{E}_x\left[\sigma_{jk}\left(X_a,v,\Gamma\right)\frac{\partial}{\partial x^j}\tilde{w}_{\lambda,a}\left(X_a,\Gamma\right)\mathcal{E}_{\lambda,a}\left(\Gamma\right)dB_a^k\right]=0.$$

Considering the following limit

$$\lim_{h\downarrow 0}\frac{1}{h}\sup_{v\in\mathcal{V}}\left\{\int_0^h d\tau\ \mathbb{E}_x\left[\left(\frac{\partial}{\partial a}-\bar{\mathcal{H}}_x^v\left(\Gamma\right)-\lambda\right)\tilde{w}_{\lambda,a_0+\tau}\left(X_\tau,\Gamma\right)\mathcal{E}_{\lambda,\tau}\left(\Gamma\right)\right]\right\}=0,$$

where $h=a-a_0$, we have

$$\begin{cases}\dfrac{\partial}{\partial a}\tilde{w}_{\lambda,a}\left(x,\Gamma\right)-\inf\limits_{v\in\mathcal{V}}\left\{\left[\bar{\mathcal{H}}_x^v\left(\Gamma\right)+\lambda\right]\tilde{w}_{\lambda,a}\left(x,\Gamma\right)\right\}=0\\ \tilde{w}_{\lambda,\alpha}\left(x,\Gamma\right)=F\left(x,\Gamma\right)\end{cases}. \tag{C.4}$$

Stationary control

For the appropriate functions $f\left(x,v,\Gamma\right)$ and $\varphi\left(x,\Gamma\right)$, the value function can be generalized such that

$$\hat{w}_{\lambda,a}\left(x\right)=\sup_{v\in\mathcal{V}}\left\{\mathbb{E}_x\left[\varphi\left(X_s\right)\mathcal{E}_{\lambda,a}\left(\Gamma\right)+\int_0^{\alpha-a}ds\ f\left(X_s,v,\Gamma\right)\mathcal{E}_{\lambda,s}\left(\Gamma\right)\right]\right\}.$$

The HJB equation then becomes

$$\begin{cases}\dfrac{\partial}{\partial a}\hat{w}_{\lambda,a}\left(x,\Gamma\right)-\inf\limits_{v\in\mathcal{V}}\left\{\left[\bar{\mathcal{H}}_x^v\left(\Gamma\right)+\lambda\right]\hat{w}_{\lambda,a}\left(x,\Gamma\right)-f\left(x,v,\Gamma\right)\right\}=0\\ \hat{w}_{\lambda,\alpha}\left(x,\Gamma\right)=\varphi\left(x,\Gamma\right).\end{cases} \tag{C.5}$$

Setting $\alpha=\infty$, $f\left(x,v,\Gamma\right)=F\left(y,\Gamma\right)$, and using

$$\tilde{\psi}_\lambda\left(x,\Gamma\right)=\lim_{\alpha\uparrow\infty}\hat{w}_{\lambda,a}\left(x,\Gamma\right)=\sup_{v\in V}\left\{\int_0^\infty ds\ \mathbb{E}_x\left[F\left(X_s,\Gamma\right)\mathcal{E}_{\lambda,s}\left(\Gamma\right)\right]\right\} \tag{C.6}$$

as another value function, the function satisfies the following HJB equation:

$$F\left(x,\Gamma\right)-\inf_{v\in\mathcal{V}}\left\{\left[\bar{\mathcal{H}}_x^v\left(\Gamma\right)+\lambda\right]\tilde{\psi}_\lambda\left(x,\Gamma\right)\right\}=0, \tag{C.7}$$

because of Eq. (C.5) . The control providing this kind of value function is called stationary control.

Text D

Analysis of the resource acquisition competition model

Assumptions of the specific model are made such that

$$y\in\left(0,x^*\right) \tag{D.1}$$

$$q=0 \tag{D.2}$$

$$g_j\left(y,v,\Gamma_{t,x}\right)\equiv\left(b_1-\Gamma_{t,x}\right)y, \tag{D.3}$$

$$c_{jj'}\left(y,v,\Gamma_{t,x}\right)\equiv\sigma_1^2y^2, \tag{D.4}$$

$$\mu\left(y,v,\Gamma_{t,x}\right)\equiv\mu_0, \tag{D.5}$$

$$\gamma\left(a,y\right)\equiv\gamma_0, \tag{D.6}$$

$$S_{\mathrm{S}}\left(a\right)\equiv\mathbb{I}_{\{a\le a^*\}}\exp\left\{-\mu_0 a\right\}\qquad a^*:=\inf_a\left\{a>0\middle|X_a\ge x^*\right\}, \tag{D.7}$$

and

$$F_{\mathrm{S}}(y) := \begin{cases} \phi(y) & y = x^* \\ 0 & y \neq x^*. \end{cases} \tag{D.8}$$

$$\mathcal{H}_y^{1,v} = \frac{\partial}{\partial y} y.$$

In the specific model ($d = M = 1, \alpha = \infty$), the characteristic equation Eq. (B.24) becomes

$$\begin{aligned} \Psi_\lambda(x) &= \psi_\lambda^*(x) - \frac{I_\lambda^*(x) \int_A d\xi \ \psi_\lambda^*(\xi) \mathcal{H}_\xi^{1,v} \hat{P}^*(\xi)}{1 + \int_A d\xi \ I_\lambda^*(\xi) \mathcal{H}_\xi^{1,v} \hat{P}^*(\xi)} \\ &= \psi_\lambda^*(x) + \frac{I_\lambda^*(x) \int_A d\xi \ \psi_\lambda^*(\xi) \frac{\partial}{\partial \xi} \xi \hat{P}^*(\xi)}{1 - \int_A d\xi \ I_\lambda^*(\xi) \frac{\partial}{\partial \xi} \xi \hat{P}^*(\xi)} \\ &= \psi_\lambda^*(x) - \frac{I_\lambda^*(x) \int_A d\xi \ \left(\frac{\partial}{\partial \xi} \psi_\lambda^*(\xi)\right) \xi \hat{P}^*(\xi)}{1 + \int_A d\xi \ \left(\frac{\partial}{\partial \xi} I_\lambda^*(\xi)\right) \xi \hat{P}^*(\xi)}, \end{aligned} \tag{D.9}$$

from

$$\left[\psi_\lambda^*(\xi) \xi \hat{P}^*(\xi)\right]_{\xi=0}^{\xi=x^*} = 0.$$

Using the configuration from the previous section, we have the following lemma.

Lemma 1. *Under Eqs.*(D.1)-(D.8)*, the following equality holds.*

$$\frac{\partial}{\partial x} I_\lambda^*(x) = -\frac{\gamma_0}{\phi(x^*)(\lambda + \mu_0)} \frac{\partial}{\partial x} \psi_\lambda^*(x) \tag{D.10}$$

Proof. It follows from Eqs.(D.6)-(D.8), (B.19), and (B.20) that

$$\begin{aligned} \psi_\lambda^*(x) &= \int_0^\infty da \ \exp\{-\lambda a\} \mathbb{E}_x [F_S(X_a) S_S(a)] \\ &= \int_0^\infty da \exp\{-\lambda a\} \mathbb{E}_x [F_{\mathrm{S}}(X_a) S_{\mathrm{S}}(a)] \\ &= \mathbb{E}_x \left[\int_0^\infty da \exp\{-\lambda a\} F_{\mathrm{S}}(X_a) S_{\mathrm{S}}(a)\right] \\ &= \mathbb{E}_x \left[\int_{a^*}^\infty da \exp\{-\lambda a\} F_{\mathrm{S}}(X_a) S_{\mathrm{S}}(a)\right] \leftarrow \text{because of Eqs.(29) and (30)} \\ &= \mathbb{E}_x \left[\exp\{-\lambda a^*\} \int_0^\infty d\tau \exp\{-\lambda \tau\} F_{\mathrm{S}}(X_{\tau + a^*}) S_{\mathrm{S}}(\tau + a^*)\right] \\ &= \mathbb{E}_x \left[\exp\{-\lambda a^*\} S_{\mathrm{S}}(a^*) \mathbb{E}_{x^*} \left[\int_0^\infty d\tau \exp\{-\lambda \tau\} F_{\mathrm{S}}(X_\tau) S_{\mathrm{S}}(\tau)\right]\right] \\ &= \mathbb{E}_x [\exp\{-\lambda a^*\} S_{\mathrm{S}}(a^*)] \mathbb{E}_{x^*} \left[\int_0^\infty d\tau \exp\{-\lambda \tau\} F_{\mathrm{S}}(X_\tau) S_{\mathrm{S}}(\tau)\right] \\ &= \mathbb{E}_x [\exp\{-\lambda a^*\} S_{\mathrm{S}}(a^*)] \phi(x^*) \qquad \uparrow \text{using strong Markov property} \\ &= \phi(x^*) \mathbb{E}_x [\exp\{-(\lambda + \mu_0) a^*\}] \end{aligned}$$

and

$$\begin{aligned} I_\lambda^*(x) &= \int_0^\infty da\ \exp\{-\lambda a\}\,\mathbb{E}_x\left[\gamma_0 S_S(a)\right] \\ &= \gamma_0 \mathbb{E}_x\left[\int_0^{a^*} da\ \exp\{-(\lambda+\mu_0)a\}\right] \\ &= \frac{\gamma_0}{\lambda+\mu_0}\left(1-\mathbb{E}_x\left[\exp\{-(\lambda+\mu_0)a^*\}\right]\right) \end{aligned}$$

in semelparous species (Cf. [3,4]). □

We rewrite Eq. (D.9) such that

$$1 = \Psi_\lambda = \psi_\lambda^*(x) - \frac{I_\lambda(x)\int_A d\xi\ \left(\frac{\partial}{\partial\xi}\psi_\lambda^*(\xi)\right)\xi\hat{P}^*(\xi)}{1-\gamma_0\int_A d\xi\frac{\left(\frac{\partial}{\partial\xi}\psi_\lambda^*(\xi)\right)\xi\hat{P}^*(\xi)}{\phi(x^*)(\lambda+\mu_0)}} \tag{D.11}$$

$$= \psi_\lambda^*(x) - \frac{\rho_\lambda I_\lambda(x)\int_A d\xi\ \psi_\lambda^*(\xi)\,\hat{P}^*(\xi)}{1-\frac{\gamma_0\rho_\lambda}{\phi(x^*)(\lambda+\mu_0)}\int_A d\xi\psi_\lambda^*(\xi)\,\hat{P}^*(\xi)} \tag{D.12}$$

Additionally, we can compute a part of the equation above by using Eq. (23) such that

$$\begin{aligned} &\int_A d\xi\ \psi_\lambda^*(\xi)\,\hat{P}^*(\xi) \\ &= \int_A d\xi \int_0^\infty da \int_0^\infty ds\ \exp\{-\lambda(a+s)\}\,u_a^*(\xi)\exp\{\lambda s\}\,P^*(s,\xi) \\ &= \int_A d\xi \int_0^\infty da \exp\{-\lambda a\}\int_0^a ds\ u_{a-s}^*(\xi)\exp\{\lambda s\}\,P^*(s,\xi) \\ &= \int_A d\xi \int_0^\infty da \exp\{-\lambda a\}\int_0^a ds\ u_{a-s}^*(\xi)\exp\{\lambda s\}\,P^*(s,\xi) \\ &= \frac{\Gamma_x^*}{I_0^*(x)}\int_A d\xi \int_0^a ds\ \exp\{-\lambda(a-s)\}\int_A dy\ F_{\mathrm{S}}(y)\,K_{a-s}^*(\xi\to y)\,K_s^*(x\to\xi) \\ &= \frac{\Gamma_x^*}{I_0^*(x)}\int_0^\infty da \int_0^a ds\ \exp\{-\lambda(a-s)\}\int_A dy\ F_{\mathrm{S}}(y)\int_A d\xi K_{a-s}^*(\xi\to y)\,K_s^*(x\to\xi)\,. \end{aligned} \tag{D.13}$$

From the semigroup property of K_{a-s}^* and K_s^*, we can apply the Chapman-Kolmogorov equation to derive

$$\int_A d\xi K_{a-s}^*(\xi\to y)\,K_s^*(x\to\xi) = K_{a-s+s}^*(x\to y) \tag{D.14}$$

$$= K_a^*(x\to y)\,, \tag{D.15}$$

which yields

$$\begin{aligned} \int_A d\xi\ \psi_\lambda^*(\xi)\,\hat{P}^*(\xi) &= \frac{\Gamma_x^*}{I_0^*(x)}\int_0^\infty da\exp\{-\lambda a\}\int_0^a ds\ \exp\{\lambda s\}\int_A dy\ F_{\mathrm{S}}(y)\,K_a^*(x\to y) \\ &= \frac{\Gamma_x^*}{I_0^*(x)}\int_0^\infty da\exp\{-\lambda a\}\int_0^a ds\ \exp\{\lambda s\}\ u_a^*(x) \\ &= \frac{\Gamma_x^*}{\lambda I_0^*(x)}\int_0^\infty da\exp\{-\lambda a\}\left(\exp\{\lambda a\}-1\right)u_a^*(x) \\ &= \frac{\Gamma_x^*}{\lambda I_0^*(x)}\left(1-\psi_\lambda^*(x)\right). \end{aligned} \tag{D.16}$$

Substituting Eq. (D.16) into Eq. (D.12), we obtain

$$\Psi_\lambda = \psi_\lambda^*(x) - \frac{\rho_\lambda \Gamma_x^* I_\lambda(x)}{\lambda I_0^*(x)} \frac{(1-\psi_\lambda^*(x))}{1 - \frac{\Gamma_x^*}{\lambda I_0^*(x)} \frac{\gamma_0 \rho_\lambda}{\phi(x^*)(\lambda+\mu_0)} (1-\psi_\lambda^*(x))} \quad \text{(D.17)}$$

$$= \psi_\lambda^*(x) - \frac{\rho_\lambda \Gamma_x^* I_\lambda(x)\, \phi(x^*)(\lambda+\mu_0)(1-\psi_\lambda^*(x))}{\lambda I_0^*(x)\, \phi(x^*)(\lambda+\mu_0) - \gamma_0 \rho_\lambda \Gamma_x^* (1-\psi_\lambda^*(x))} \quad \text{(D.18)}$$

$$= \psi_\lambda^*(x) - \frac{\mu_0 (\phi(x^*) - \psi_\lambda^*(x)) \rho_\lambda \Gamma_x^* (1-\psi_\lambda^*(x))}{\lambda(\mu_0+\lambda)(\phi(x^*)-1) - \mu_0 \rho_\lambda \Gamma_x^* (1-\psi_\lambda^*(x))}. \quad \text{(D.19)}$$

From $\Psi_{\lambda^\dagger} = 1$, we have

$$1 = \psi_\lambda^*(x) - \frac{\mu_0 (\phi(x^*) - \psi_\lambda^*(x)) \rho_\lambda \Gamma_x^* (1-\psi_\lambda^*(x))}{\lambda(\mu_0+\lambda)(\phi(x^*)-1) - \mu_0 \rho_\lambda \Gamma_x^* (1-\psi_\lambda^*(x))}, \quad \text{(D.20)}$$

that is,

$$1 - \psi_\lambda^*(x) = -\frac{\mu_0 (\phi(x^*) - \psi_\lambda^*(x)) \rho_\lambda \Gamma_x^* (1-\psi_\lambda^*(x))}{\lambda(\mu_0+\lambda)(\phi(x^*)-1) - \mu_0 \rho_\lambda \Gamma_x^* (1-\psi_\lambda^*(x))}. \quad \text{(D.21)}$$

Dividing the above equation by $1 - \psi_\lambda^*$ gives

$$1 = -\frac{\mu_0 (\phi(x^*) - \psi_\lambda^*(x)) \rho_\lambda \Gamma_x^*}{\lambda(\mu_0+\lambda)(\phi(x^*)-1) - \mu_0 \rho_\lambda \Gamma_x^* (1-\psi_\lambda^*(x))}. \quad \text{(D.22)}$$

Therefore, we obtain

$$\lambda(\mu_0+\lambda)(\phi(x^*)-1) - \mu_0 \rho_\lambda \Gamma_x^* (1-\psi_\lambda^*(x)) = -\mu_0 (\phi(x^*) - \psi_\lambda^*(x)) \rho_\lambda \Gamma_x^* \quad \text{(D.23)}$$

$$\lambda(\mu_0+\lambda) + \mu_0 \rho_\lambda \Gamma_x^* = 0. \quad \text{(D.24)}$$

Because ρ_λ satisfies

$$\rho_\lambda \left(\frac{\sigma_1^2}{2} \rho_\lambda + b_1 - \Gamma_x^* - \frac{\sigma_1^2}{2} \right) - \mu_0 - \lambda = 0, \quad \text{(D.25)}$$

the characteristic equation becomes

$$\lambda \left(\frac{\sigma_1^2}{2} \rho_\lambda + b_1 - \Gamma_x^* - \frac{\sigma_1^2}{2} \right) + \mu_0 \Gamma_x^* = 0 \quad \text{(D.26)}$$

$$\lambda \left(\sqrt{\left(1 - \frac{2(b_1 - \Gamma_x^*)}{\sigma_1^2}\right)^2 + \frac{8\mu_0}{\sigma_1^2} + \frac{8\lambda}{\sigma_1^2}} - \left(1 - \frac{2(b_1 - \Gamma_x^*)}{\sigma_1^2}\right) \right) + \frac{4\mu_0 \Gamma_x^*}{\sigma_1^2} = 0. \quad \text{(D.27)}$$

Because this equation is monotonically increasing in λ, it does not have positive real roots. Moreover, rewriting the equation as

$$\sqrt{\left(1 - \frac{2(b_1 - \Gamma_x^*)}{\sigma_1^2}\right)^2 + \frac{8\mu_0}{\sigma_1^2} + \frac{8\lambda}{\sigma_1^2}} = 1 - \frac{2(b_1 - \Gamma_x^*)}{\sigma_1^2} - \frac{4\mu_0 \Gamma_x^*}{\lambda \sigma_1^2} \quad \text{(D.28)}$$

$$\left(1 - \frac{2(b_1 - \Gamma_x^*)}{\sigma_1^2}\right)^2 + \frac{8\mu_0}{\sigma_1^2} + \frac{8\lambda}{\sigma_1^2} = \left(1 - \frac{2(b_1 - \Gamma_x^*)}{\sigma_1^2} - \frac{4\mu_0 \Gamma_x^*}{\lambda \sigma_1^2}\right)^2 \quad \text{(D.29)}$$

$$\frac{8\mu_0}{\sigma_1^2} + \frac{8\lambda}{\sigma_1^2} = -2 \left(\frac{4\mu_0 \Gamma_x^*}{\lambda \sigma_1^2} \right) \left(1 - \frac{2(b_1 - \Gamma_x^*)}{\sigma_1^2}\right) + \left(\frac{4\mu_0 \Gamma_x^*}{\lambda \sigma_1^2} \right)^2 \quad \text{(D.30)}$$

$$\mu_0 + \lambda = -\frac{\mu_0 \Gamma_x^*}{\lambda} \left(1 - \frac{2(b_1 - \Gamma_x^*)}{\sigma_1^2}\right) + \frac{2}{\sigma_1^2} \left(\frac{\mu_0 \Gamma_x^*}{\lambda} \right)^2 \quad \text{(D.31)}$$

$$\mu_0 \lambda^2 + \lambda^3 = -\mu_0 \Gamma_x^* \left(1 - \frac{2(b_1 - \Gamma_x^*)}{\sigma_1^2}\right) \lambda + 2 \left(\frac{\mu_0 \Gamma_x^*}{\sigma_1} \right)^2, \quad \text{(D.32)}$$

the characteristic function is

$$\lambda^3 + \mu_0 \lambda^2 + \mu_0 \Gamma_x^* \left(1 - \frac{2\left(b_1 - \Gamma_x^*\right)}{\sigma_1^2}\right) \lambda - 2\left(\frac{\mu_0 \Gamma_x^*}{\sigma_0}\right)^2 = 0. \tag{D.33}$$

Although Eq. (D.33) has a positive root r, it does not share the roots of Eq. (D.27). If Eq. (D.33) does not have complex roots because it has a non-negative real part, the equilibrium is stable. To prove this, we use *reductio ad absurdum*. Suppose, on the contrary, that $c \pm id$ with $c \geq 0$ are roots of Eq. (D.33). The equation can then be written as

$$(\lambda - r)(\lambda - c + id)(\lambda - c - id) = 0 \tag{D.34}$$

$$\lambda^3 - (2c + r)\lambda^2 + \left(2cr + c^2 + d^2\right)\lambda - r\left(c^2 + d^2\right) = 0. \tag{D.35}$$

$2c + r$ is positive; hence, this equation does not correspond to Eq. (D.33). Therefore, Eq. (D.27) only has roots with negative real parts.

Text E

The mature age density of the resource acquisition competition model

The mature age density of the resource acquisition competition model in each equilibrium $\mathbb{A}_{\mathrm{S}}(a, \Gamma)$ can be obtained using Eq. (40) and its inverse transform such that

$$\begin{aligned} \mathbb{A}_{\mathrm{S}}(a, \Gamma) &= \lim_{\beta \uparrow \infty} \frac{1}{2\pi i} \int_{c-i\beta}^{c+i\beta} d\lambda \exp\{\lambda a\} \frac{\psi_{\mathrm{S}\lambda}(x, \Gamma)}{\psi_{\mathrm{S}0}(x, \Gamma)} \\ &= \frac{\phi(x^*)}{\psi_{\mathrm{S}0}(x, \Gamma)} \frac{\ln \frac{x^*}{x}}{\sqrt{2\pi {\sigma_1}^2 a^3}} \exp\left\{ -\frac{\left[\ln \frac{x^*}{x} - \left(b_1 - \Gamma - \frac{{\sigma_1}^2}{2}\right) a\right]^2}{2{\sigma_1}^2 a} - \mu_0 a \right\}. \end{aligned} \tag{E.1}$$

The details of the proof are provided below: **Lemma.** For the positive constants K_1, K_2 and K_3, the following holds

$$\int_0^\infty \frac{1}{\sqrt{a^3}} \exp\left\{ -\left(\sqrt{K_1}\sqrt{a} - \frac{\sqrt{K_3}}{\sqrt{a}}\right)^2 \right\} da = \frac{\sqrt{\pi}}{\sqrt{K_3}}. \tag{E.2}$$

Proof. Because

$$\begin{aligned} &\int_0^\infty da \; \frac{1}{\sqrt{a^3}} \exp\left\{ -\left(\sqrt{K_1}\sqrt{a} - \frac{\sqrt{K_3}}{\sqrt{a}}\right)^2 \right\} \\ &= \int_0^\infty \frac{1}{t^3} \exp\left\{ -\left(\sqrt{K_1} t - \frac{\sqrt{K_3}}{t}\right)^2 \right\} \cdot 2t \; dt \\ &= 2\int_0^\infty \frac{1}{t^2} \exp\left\{ -\left(\sqrt{K_1} t - \frac{\sqrt{K_3}}{t}\right)^2 \right\} dt, \end{aligned}$$

it suffices to prove that

$$\int_0^\infty dt \; \frac{1}{t^2} \exp\{-(\sqrt{K_1} t - \frac{\sqrt{K_3}}{t})^2\} = \frac{\sqrt{\pi}}{2\sqrt{K_3}}.$$

Through the variable transformation $t = \frac{\sqrt{K_3}}{\sqrt{K_1}} \frac{1}{s}$, we see that

$$\frac{1}{t^2} = \frac{K_1}{K_3} s^2, \quad -\left(\sqrt{K_1} t - \frac{\sqrt{K_3}}{t}\right)^2 = -\left(\frac{\sqrt{K_3}}{s} - \sqrt{K_1} s\right)^2 = -\left(\sqrt{K_1} s - \frac{\sqrt{K_3}}{s}\right)^2$$

and

$$\begin{aligned}
&\int_0^\infty dt\ \frac{1}{t^2}\exp\left\{-\left(\sqrt{K_1}t-\frac{\sqrt{K_3}}{t}\right)^2\right\}\\
&=\int_\infty^0 ds\ \frac{K_1}{K_3}s^2\exp\left\{-\left(\sqrt{K_1}s-\frac{\sqrt{K_3}}{s}\right)^2\right\}\left(-\frac{\sqrt{K_3}}{\sqrt{K_1}}\frac{1}{s^2}\right)\\
&=-\int_\infty^0 ds\ \frac{\sqrt{K_1}}{\sqrt{K_3}}\exp\left\{-\left(\sqrt{K_1}s-\frac{\sqrt{K_3}}{s}\right)^2\right\}\\
&=\int_0^\infty ds\ \frac{\sqrt{K_1}}{\sqrt{K_3}}\exp\left\{-\left(\sqrt{K_1}s-\frac{\sqrt{K_3}}{s}\right)^2\right\}\\
&=\int_0^\infty dt\ \frac{\sqrt{K_1}}{\sqrt{K_3}}\exp\left\{-\left(\sqrt{K_1}t-\frac{\sqrt{K_3}}{t}\right)^2\right\}.
\end{aligned}\tag{E.3}$$

On the other hand, the following holds:

$$\begin{aligned}
&\int_0^\infty dt\ \frac{1}{t^2}\exp\left\{-\left(\sqrt{K_1}t-\frac{\sqrt{K_3}}{t}\right)^2\right\}+\int_0^\infty dt\ \frac{\sqrt{K_1}}{\sqrt{K_3}}\exp\left\{-\left(\sqrt{K_1}t-\frac{\sqrt{K_3}}{t}\right)^2\right\}\\
=&\int_0^\infty dt\ \left(\frac{1}{t^2}+\frac{\sqrt{K_1}}{\sqrt{K_3}}\right)\exp\left\{-\left(\sqrt{K_1}t-\frac{\sqrt{K_3}}{t}\right)^2\right\}\\
=&\frac{1}{\sqrt{K_3}}\int_0^\infty dt\ \left(\sqrt{K_1}t-\frac{\sqrt{K_3}}{t}\right)'\exp\left\{-\left(\sqrt{K_1}t-\frac{\sqrt{K_3}}{t}\right)^2\right\}\\
=&\frac{1}{\sqrt{K_3}}\int_{-\infty}^\infty du\ \ \exp\{-u^2\}=\frac{\sqrt{\pi}}{\sqrt{K_3}}.
\end{aligned}\tag{E.4}$$

It follows from Eqs.(E.3) and (E.4) that

$$\begin{aligned}
&\int_0^\infty dt\ \frac{1}{t^2}\exp\{-(\sqrt{K_1}t-\frac{\sqrt{K_3}}{t})^2\}=\int_0^\infty dt\ \frac{\sqrt{K_1}}{\sqrt{K_3}}\exp\{-(\sqrt{K_1}t-\frac{\sqrt{K_3}}{t})^2\}\\
&=\frac{\sqrt{\pi}}{2\sqrt{K_3}}.
\end{aligned}$$

Therefore, we obtain

$$\int_0^\infty \frac{1}{\sqrt{a^3}}\exp\left\{-\left(\sqrt{K_1}\sqrt{a}-\frac{\sqrt{K_3}}{\sqrt{a}}\right)^2\right\}da=2\cdot\frac{\sqrt{\pi}}{2\sqrt{K_3}}=\frac{\sqrt{\pi}}{\sqrt{K_3}}.$$

This completes the proof. □

Multiplying (E.2) by $\frac{\log\frac{x^*}{x}}{\sqrt{2\pi\sigma_0^2}}\exp\{-K_2-2\sqrt{K_1K_3}\}$ gives

$$\int_0^\infty \frac{\log\frac{x^*}{x}}{\sqrt{2\pi\sigma_0^2a^3}}\exp\left\{-K_1a-K_2-\frac{K_3}{a}\right\}da=\frac{\log\frac{x^*}{x}}{\sqrt{2K_3\sigma_0^2}}\exp\{-K_2-2\sqrt{K_1K_3}\}.\tag{E.5}$$

We substitute

$$K_1=\frac{(b-\frac{\sigma_0^2}{2})^2}{2\sigma_0^2}+\mu_0+\lambda,\ K_2=-\frac{\log\frac{x^*}{x}(b-\frac{\sigma_0^2}{2})}{\sigma_0^2},\ K_3=\frac{(\log\frac{x^*}{x})^2}{2\sigma_0^2}$$

into Eq. (E.5) . Then, we have $\frac{\log \frac{x^*}{x}}{\sqrt{2K_3\sigma_0^2}} = 1$ and

$$\begin{aligned}
-K_2 - 2\sqrt{K_1K_3} &= \frac{\log \frac{x^*}{x}(b - \frac{\sigma_0^2}{2})}{\sigma_0^2} - \frac{\log \frac{x^*}{x}}{\sigma_0}\sqrt{\frac{(b - \frac{\sigma_0^2}{2})^2}{\sigma_0^2} + 2(\mu_0 + \lambda)} \\
&= \log \frac{x^*}{x}\left\{\frac{1}{2}\left(\frac{2b}{\sigma_0^2} - 1\right) - \frac{1}{2}\sqrt{\left(\frac{2b}{\sigma_0^2} - 1\right)^2 + \frac{8(\mu_0 + \lambda)}{\sigma_0^2}}\right\} \\
&= \log \frac{x}{x^*}\left\{\frac{1}{2}\left(1 - \frac{2b}{\sigma_0^2}\right) + \frac{1}{2}\sqrt{\left(1 - \frac{2b}{\sigma_0^2}\right)^2 + \frac{8(\mu_0 + \lambda)}{\sigma_0^2}}\right\} = \rho_\lambda \log \frac{x}{x^*},
\end{aligned}$$

which implies

$$\int_0^\infty \frac{\log \frac{x^*}{x}}{\sqrt{2\pi\sigma_0^2 a^3}} \exp\left\{-K_1 a - K_2 - \frac{K_3}{a}\right\} da = \exp\left\{\rho_\lambda \log \frac{x}{x^*}\right\} = \left(\frac{x}{x^*}\right)^{\rho_\lambda}.$$

Text F

Analysis of the optimal utilization

As preparation for analyzing the optimal utilization, the adjoint Hamiltonian of Eqs. (33) and (46) becomes

$$\bar{\mathcal{H}}_x^v = -\left[\theta_1(1-v) + \theta_2 v\right] x\frac{d}{dx} - \frac{1}{2}\left[(\sigma_1(1-v))^2 + (\sigma_2 v)^2\right] x^2 \frac{d^2}{dx^2} + \mu_0. \tag{F.1}$$

Let $\varphi(x) \in C^2(\mathbb{R}_+)$ provide an extreme value of $\left[\bar{\mathcal{H}}_x^v + \lambda\right]\varphi(x)$ with respect to v such that

$$\frac{\partial}{\partial v}\left[\bar{\mathcal{H}}_x^v + \lambda\right]\varphi(x)\Big|_{v=v^\dagger} = 0. \tag{F.2}$$

Then, the value satisfies

$$v^\dagger(\varphi(x)) = \frac{\sigma_1^2}{\sigma_1^2 + \sigma_2^2} + \frac{(\theta_1 - \theta_2)\frac{d}{dx}\varphi(x)}{(\sigma_1^2 + \sigma_2^2)\, x \frac{d^2}{dx^2}\varphi(x)}, \tag{F.3}$$

and we obtain a nonlinear operator by substituting Eqs.(F.3) into (F.1) as follows:

$$\begin{aligned}
H_\lambda(\varphi(x)) &:= \left[\bar{\mathcal{H}}_x^v + \lambda\right]\varphi(x)\Big|_{v=v^\dagger} \\
&= -\left[\frac{\theta_1\sigma_2^2 + \theta_2\sigma_1^2}{\sigma_1^2 + \sigma_2^2}\right] x\frac{\partial}{\partial x}\varphi(x) - \frac{1}{2}\frac{\sigma_1^2\sigma_2^2}{\sigma_1^2 + \sigma_2^2} x^2 \frac{\partial^2}{\partial x^2}\varphi(x) \\
&\quad + \frac{1}{2}\frac{(\theta_1 - \theta_2)^2\left(\frac{\partial}{\partial x}\varphi(x)\right)^2}{(\sigma_1^2 + \sigma_2^2)\frac{\partial^2}{\partial x^2}\varphi(x)} + (\mu_0 + \lambda)\varphi(x).
\end{aligned} \tag{F.4}$$

Suppose that

$$\tilde{\psi}_\lambda(x, \Gamma) = cx^\rho \qquad c \neq 0. \tag{F.5}$$

Substituting this function into Eq. (F.4) as follows,

$$\begin{aligned}
&H_\lambda(cx^\rho) = \\
&\left[-\left(\frac{\theta_1\sigma_2^2 + \theta_2\sigma_1^2}{\sigma_1^2 + \sigma_2^2}\right)\rho + \frac{1}{2}\frac{\sigma_1^2\sigma_2^2}{\sigma_1^2 + \sigma_2^2}\rho(1-\rho) - \frac{1}{2}\frac{(\theta_1 - \theta_2)^2\rho}{(\sigma_1^2 + \sigma_2^2)(1-\rho)} + (\mu_0 + \lambda)\right] \\
&\times cx^\rho = 0,
\end{aligned} \tag{F.6}$$

and ρ should satisfy $\rho > 0$ and the equation above. In addition, because $\tilde{\psi}_\lambda(x, \Gamma)$ should satisfy Eq. (D.8) , the solution becomes

$$\tilde{\psi}_\lambda(x, \Gamma) = \phi(x^*) \left(\frac{x}{x^*}\right)^\rho. \tag{F.7}$$

From Eqs.(11) and (38), the IRNI of the r-strategy $\tilde{\lambda}_\mathrm{r}(\rho^*)$ becomes

$$\tilde{\lambda}_\mathrm{r}(\rho^*) = \begin{cases} \left[b_1 - \frac{\sigma_1^2}{2}(1-\rho^*)\right]\rho^* - \mu_0 & \text{if} \quad \tilde{v}_\mathrm{r}(\rho^*) = 0 \\ \frac{\rho^*}{\sigma_1^2+\sigma_2^2}\left[b_1\sigma_2^2 + b_2\sigma_1^2 - \frac{1}{2}\sigma_1^2\sigma_2^2(1-\rho^*) + \frac{1}{2}\frac{(b_1-b_2)^2}{(1-\rho^*)}\right] - \mu_0 & \text{if} \quad 0 < \tilde{v}_\mathrm{r}(\rho^*) < 1 \\ \left[b_2 - \frac{\sigma_2^2}{2}(1-\rho^*)\right]\rho^* - \mu_0 & \text{if} \quad \tilde{v}_\mathrm{r}(\rho^*) = 1 \end{cases}, \tag{F.8}$$

Incidentally, the IRNI with respect to the arbitrary constant strategy v becomes

$$\lambda^* = \left[b_1(1-v) + b_2 v - \frac{1}{2}\left(\sigma_1^2(1-v)^2 + \sigma_2^2 v^2\right)(1-\rho^*)\right]\rho^* - \mu_0. \tag{F.9}$$

On the other hand, the non-trivial equilibrium of the K-strategy satisfies Eq. (15), which follows

$$\tilde{\Gamma} = \begin{cases} \frac{1}{\kappa}\left[b_1 - \frac{{\sigma_1}^2}{2}(1-\rho^*) - \frac{\mu_0}{\rho^*}\right] & \text{if} \quad \tilde{v}_\mathrm{K}(\rho^*) = 0 \\ I_1 - I_2 & \text{if} \quad 0 < \tilde{v}_\mathrm{K}(\rho^*) < 1 \\ \frac{1}{1-\kappa}\left[b_2 - \frac{{\sigma_2}^2}{2}(1-\rho^*) - \frac{\mu_0}{\rho^*}\right] & \text{if} \quad \tilde{v}_\mathrm{K}(\rho^*) = 1, \end{cases} \tag{F.10}$$

where

$$I_1 := \frac{(1-\rho^*)\left((1-\kappa){\sigma_1}^2 + \kappa{\sigma_2}^2\right)}{(1-2\kappa)^2} - \frac{b_1-b_2}{1-2\kappa}$$

$$I_2 := \sqrt{{I_1}^2 + \frac{2(1-\rho^*)}{(1-2\kappa)^2}\left[({\sigma_1}^2+{\sigma_2}^2)\frac{\mu_0}{\rho^*} + \frac{1}{2}{\sigma_1}^2{\sigma_2}^2(1-\rho^*) - \left(b_1{\sigma_2}^2 + b_2{\sigma_1}^2 + \frac{(b_1-b_2)^2}{2(1-\rho^*)}\right)\right]}. \tag{F.11}$$

Although $\tilde{\Gamma}$ when $0 < \tilde{v}_\mathrm{K}(\rho^*) < 1$ has two solutions, only the smaller one is correct because the characteristic function Eq. (40) should be a monotonically decreasing function with respect to Γ. Because the RHS of Eq. (F.6) is monotonically increasing for sufficiently large Γ, the other solution can be neglected. If $\kappa = 0.5$ on $0 < \tilde{v}_\mathrm{r}(\rho^*) = \tilde{v}_\mathrm{K}(\rho^*) < 1$, $\tilde{\Gamma}$ becomes

$$\tilde{\Gamma} = \frac{2}{\sigma_1^2+\sigma_2^2}\left[b_1\sigma_2^2 + b_2\sigma_1^2 - \frac{1}{2}\sigma_1^2\sigma_2^2(1-\rho^*) + \frac{1}{2}\frac{(b_1-b_2)^2}{(1-\rho^*)}\right] - \mu_0 \tag{F.12}$$

The adaptive strategy is obtained by substituting Eq. (F.7)into Eq. (F.3). Then, we have Eq. (54). Though the exact mathematical form of the K-strategy can be obtained by substituting Eq. (F.10) into Eq. (54), using $\tilde{\Gamma}$ provides an easier derivation of the strategy because we know that Eq. (54) is constant. Γ^* of Eq. (49) represents

$$\Gamma^* = \frac{b_1(1-v) + b_2 v - \frac{1}{2}\left(\sigma_1^2(1-v)^2 + \sigma_2^2 v^2\right)(1-\rho^*) - \frac{\mu_0}{\rho^*}}{\kappa + (1-2\kappa)v}. \tag{F.13}$$

Because the K-strategy between 0 and 1 should be the maximal value,

$$\frac{\partial}{\partial v}\Gamma^*\Big|_{v=\tilde{v}_{\mathrm{K}}} = 0, \tag{F.14}$$

we obtain the exact form as follows:

$$\tilde{v}_{\mathrm{K}} = \max\left\{\min\{J_1 + J_2, 1\}, 0\right\} \tag{F.15}$$

where

$$J_1 = \frac{-\kappa}{1-2\kappa} \tag{F.16}$$

$$J_2 = \sqrt{J_1^2 - \frac{2\left[(1-\kappa)\,b_1 - \kappa b_2\right]}{(1-2\kappa)\,(1-\rho^*)\,(\sigma_1^2+\sigma_2^2)} + \frac{2\sigma_1^2}{(1-2\kappa)\,(\sigma_1^2+\sigma_2^2)} + \frac{2\mu_0}{\rho^*\,(1-\rho^*)\,(\sigma_1^2+\sigma_2^2)}} \tag{F.17}$$

References

1. Øksendal BK. Stochastic differential equations: an introduction with applications. Springer Verlag; 2003.

2. Iannelli M. Mathematical theory of age-structured population dynamics. Giardini Editori e Stampatori in Pisa. 1995;.

3. Oizumi R, Takada T. Optimal life schedule with stochastic growth in age-size structured models: theory and an application. Journal of Theoretical Biology. 2013;323:76–89.

4. Oizumi R. Unification Theory of Optimal Life Histories and Linear Demographic Models in Internal Stochasticity. PLOS ONE. 2014;9(6):e98746.
